# Metabolomics reveals diet-derived plant polyphenols accumulate in physiological bone

**DOI:** 10.1038/s41598-019-44390-1

**Published:** 2019-05-29

**Authors:** Isabelle Alldritt, Beatrice Whitham-Agut, Miguel Sipin, Jacob Studholme, Angela Trentacoste, Jennifer A. Tripp, Maria Grazia Cappai, Peter Ditchfield, Thibaut Devièse, Robert E. M. Hedges, James S. O. McCullagh

**Affiliations:** 1 Department of Chemistry, Mansfield Road, University of Oxford, Oxford, OX1 3TA UK; 2 Institute of Archaeology, University of Oxford, Oxford, OX1 2PG UK; 3 UCL Institute of Archaeology, Gordon Square, London, WC1H 0PY UK; 4 Research Unit of Animal Nutrition, Department of Veterinary Medicine, University of Sassari, Sassari, PO Box 07100 Italy; 5 Research Laboratory for Archaeology and the History of Art, University of Oxford, Dyson Perrins Building, South Parks Road, Oxford, OX1 3QY UK

**Keywords:** Metabolomics, Bone, Element cycles, Biochemical assays

## Abstract

Plant-derived secondary metabolites consumed in the diet, especially polyphenolic compounds, are known to have a range of positive health effects. They are present in circulation after ingestion and absorption and can be sequestered into cells within particular organs, but have rarely been investigated systematically in osteological tissues. However, a small number of polyphenols and similar molecules are known to bind to bone. For example alizarin, a plant derived anthraquinone and tetracycline (a naturally occurring antibiotic), are both absorbed into bone from circulation during bone formation and are used to monitor mineralization in osteological studies. Both molecules have also been identified serendipitously in archaeological human bones derived from natural sources in the diet. Whether an analogous mechanism of sequestration extends to additional diet-derived plant-polyphenols has not previously been systematically studied. We investigated whether a range of diet-derived polyphenol-like compounds bind to bone using untargeted metabolomics applied to the analysis of bone extracts from pigs fed an acorn-based diet. We analysed the diet which was rich in ellagitannins, extracts from the pig bones and surrounding tissue, post-mortem. We found direct evidence of multiple polyphenolic compounds in these extracts and matched them to the diet. We also showed that these compounds were present in the bone but not surrounding tissues. We also provide data showing that a range of polyphenolic compounds bind to hydroxyapatite *in vitro*. The evidence for polyphenol sequestration into physiological bone, and the range and specificity of polyphenols in human and animal diets, raises intriguing questions about potential effects on bone formation and bone health. Further studies are needed to determine the stability of the sequestered molecules post-mortem but there is also potential for (palaeo)dietary reconstruction and forensic applications.

## Introduction

Polyphenolic compounds, and similar plant-derived secondary metabolites, are found in a wide range of foods including fruits, leaves, roots, and seeds and are an important constituent of the diet of humans and animals. They can provide antioxidant effects, scavenge free radicals and consumption is linked to a lower risk of diseases including cancer, cardiovascular disease and chronic inflammation^1,2^. Specific polyphenols also modulate a range of enzyme activities and provide selective pharmacological and therapeutic effects^3^.

Despite these positive health benefits, polyphenols are treated as xenobiotic molecules by the body and processed via phase 1 & 2 metabolism for removal from the organism. They are almost ubiquitously present as glycosylated molecules in plants but their digestion can take a number of routes depending on the molecular weight and chemical structure of the compound^4^. The absorption process often involves de-glycosylation, direct absorption or conjugation and absorption in the upper intestine or re-conjugation and absorption (frequently mediated by microbial metabolism) in the lower intestine and colon^4^. These differentiated absorption processes make it difficult to predict the form in which a specific polyphenol consumed will be present in circulation. A range of relatively recent studies have shown that polyphenols can enter various tissues and organs where they may provide tissue-specific therapeutic activities such as reducing inflammation or providing anti-tumorigenic effects^5–7^. It has been known for several hundred years that some polyphenols can bind to bone *in vivo*, although the process is still not well understood. In 1736, Belchier demonstrated that ingestion of root madder resulted in the staining of bones a red colour *in vivo*, and subsequently this was attributed to alizarin, a red-coloured, hydroxylated anthraquinone found in root madder, which is still used as a bone staining agent in contemporary ossification studies^8–10^. It has been suggested that the mechanism of binding to bone is likely to involve chelate formation with calcium ions during osteoblastic mineralization where new or remodeled bone is formed^11^.

Intriguingly, polyphenols and similar molecules have also been found in archaeological human remains on at least two occasions. In 1971, ‘purplish-red stained bones’ were discovered at a human burial site in Qumran on the Dead Sea^12^. Spectroscopic analysis determined that the plant polyphenol alizarin, most likely derived from the dietary consumption of root madder, was incorporated into the medullary cavity of the skeletal material, probably during mineralisation (ibid). This is supported by evidence of both salt and chelate binding between alizarin and calcium ions in hydroxyapatite (HAP)^13,14^. In a separate study, fluorescence signatures for tetracycline (an anti-biotic) were found in archaeological skeletal remains from an ancient Nubian cemetery, dating to 350 AD^15^. The pattern of fluorescence, localised around osteons in the bone, suggested that compact bone had actively been mineralising at the time of exposure to tetracycline^15^. Taken together these serendipitous findings in the archaeological record provide evidence that quite different diet-derived polyphenolic-like compounds can become sequestered into growing bone and survive over archaeological time.

A wide range of polyphenolic compounds are found in dietary fruits, nuts, and vegetables and we set out to investigate whether new dietary polyphenolic compounds could be identified in the bones of animals. We speculated that a general adsorption and sequestration mechanism may lead to a range of dietary-derived plant compounds being regularly incorporated into growing bone and that this could have interesting implications for a number of fields of research. The bioactive properties of polyphenols for example may specifically affect bone health, potentially providing prophylactic or therapeutic effects. In additional being able to identify plant-derived polyphenols in modern and archaeological bone would benefit ecological studies and those involved in (palaeo)dietary reconstruction using bone remains, where current methods provide almost no information about plant consumption. In order to systematically investigate a general mechanism for plant polyphenol binding to bone, we conducted an animal feeding study in which domesticated pigs were reared on a controlled diet based on raw acorns as the main ingredient, a rich source of certain types of polyphenol including ellagitannins. Pigs were chosen because they are a good animal model for human nutrition^16,17^. We developed and applied a solvent extraction protocol for sampling pig bones, followed by analysis using both targeted and untargeted ultra-high performance liquid chromatography coupled directly to high resolution Orbitrap mass spectrometry (UHPLC-MS/MS). We investigated whether dietary-derived polyphenolic compounds, or their metabolic products, could be identified in the skeletal bone extracts.

## Diet-derived Plant Polyphenols are Found in Animal Bones

**Figure 1:**
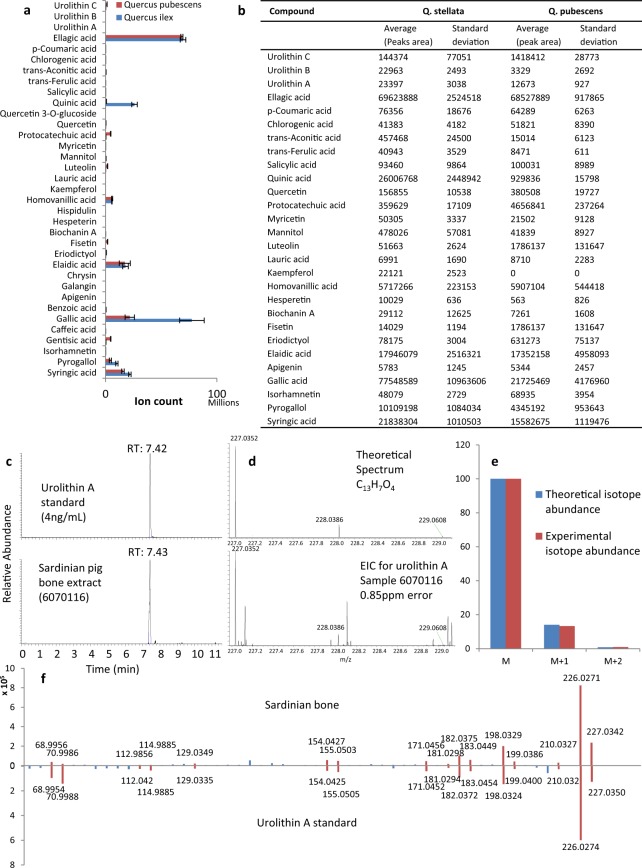
Polyphenols identified in the acorn supplement and measurements for the identification of urolithin A in pig bone extracts. (**a**) Bar graph showing magnitude of the ion count for individual polyphenols identified in the acorn extracts (error bars St. dev. n = 9/group). (**b**) Average peak areas for plant secondary metabolites identified from acorn extracts (St. dev. n = 9/compound). (**c**) Chromatographic retention time for Urolithin A in the authentic standard and Sardinian bone 6070116 is 7.42 and 7.43 mins respectively, a difference of 0.6 seconds. (**d**) The accurate *m/z* measurement of Urolithin A in the authentic standard and Sardinian bone provided a difference of 0.85 ppm. (**e**) Comparison of the relative abundance of the M, M + 1 and M + 2 isotopes for Urolithin A in the authentic standard compared to Sardinian bone showed a 99% similarity. (**f**) Comparison of the product ion spectra from the collision induced dissociation (CID) of urolithin A (precursor *m/z* 227.03) in the Sardinian extract (top) and product ion spectra from the analysis of the urolithin A standard (bottom).

Five domesticated pigs were raised in a diet-controlled environment in Sardinia on a commercial feed combined with 50% (by weight) shredded raw acorns (a mixture of Quercus ilex and Quercus pubescens). We analysed a solvent extract from the dietary acorns using untargeted metabolite profiling by UHPLC-MS/MS. A large number of compound features (9561) were detected and from these 28 polyphenols were identified by further comparison with analysis of 105 authentic plant secondary metabolite standards (Fig. 1 & Supplementary Table 2).

Next, we analysed multiple ethanol extracts from different pieces of femur bone from each of the five Sardinian pigs reared on the experimental acorn-rich diets, using the same untargeted UHPLC-MS/MS method. A bone from a commercially procured ‘Iberico ham’ was also analysed as a positive control (jamón ibérico cebo de campo hams are commercially controlled and required to have been fed a combination of acorns and grain, in accordance with Spain’s food product rules)^18^. A separate femur from a pig whose diet did not contain any acorn supplement, was also analysed as a negative control for the acorn diet supplementation (Supplementary Table 1c). Over 26,000 individual compound features were measured in the pig-bone extracts and Supplementary Fig. 1 shows a representative total ion chromatogram and ‘ion-map’ for these analyses. We analysed our 105 plant-derived authentic standards (Supplementary Table 2) in the same analytical sequence (after the bone samples) using the same UHPLC-MS/MS method (Methods). Appropriate blanks were used between each sample and the experiments repeated using fresh bone extracts on multiple occasions over a 6 month period to confirm the authenticity of the findings and to ensure no carry over or contamination compromised the results.

**Figure 2:**
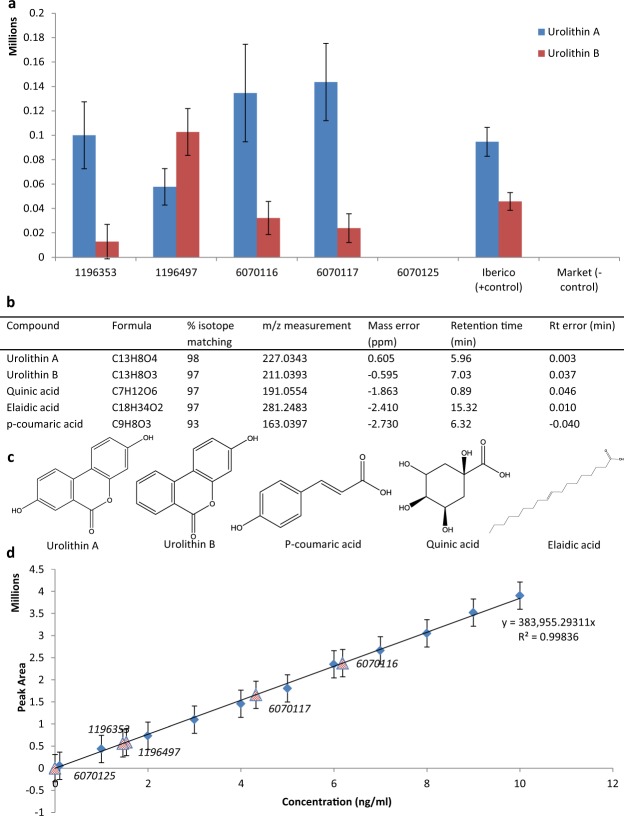
Identification of plant-derived polyphenols in animal bone. (**a**) Relative abundance (chromatographic peak area) for urolithin A and urolithin B in extracts from each of the Sardinian bones (mean and St. dev. n = 5). (**b)** Table showing the matching criteria for the identification of diet-derived compounds in bone extracts. (**c)** Structures of the five plant-derived compounds identified in pig bones from the animal feeding study. (**d**) External calibration curve showing linearity for the urolithin A peak area response to concentration over the range 10 ng/mL to 0.08 ng/mL. The peak areas generated for urolithin A in each of the Sardinian bone extracts are also plotted and labelled (red triangles) on the line of best fit.

**Figure 3:**
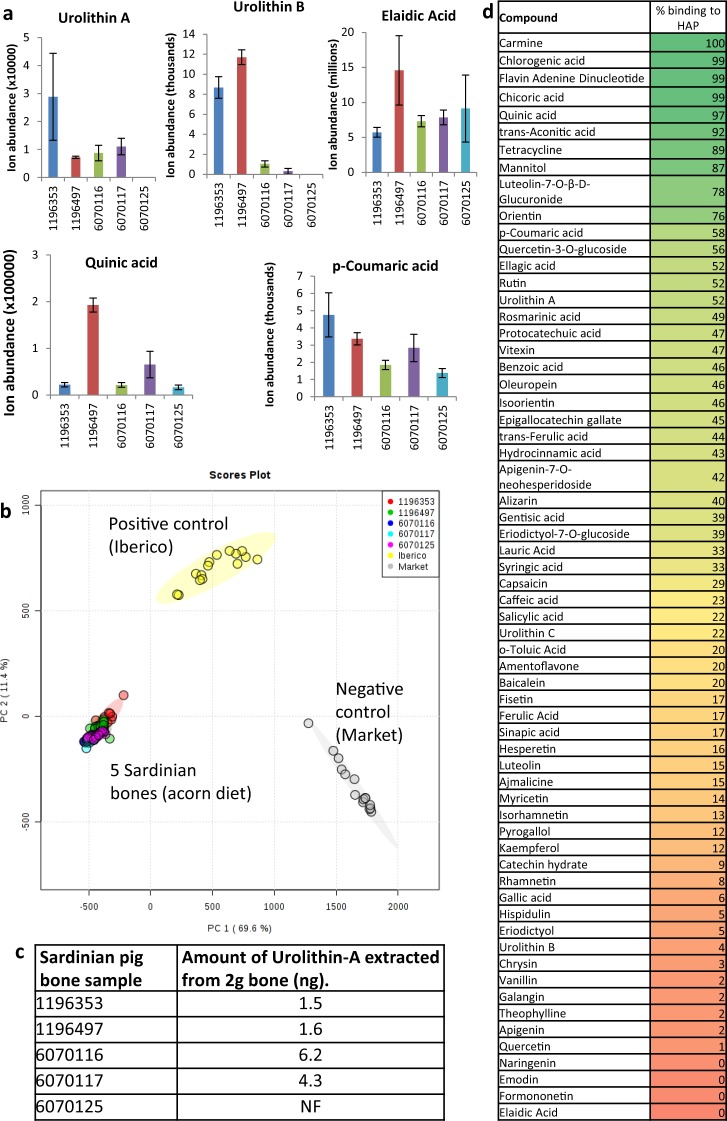
Identification of plant-derived polyphenols in bones and bone model. (**a**) Relative ion abundances for the 5 metabolites identified from our in house ‘polyphenol database’ across the 5 Sardinian bones extracts (error bars st. dev; n = 3). (**b**) PCA scores plot shows samples cluster into dietary groups based on their compound feature composition. (**c**) Table showing the calculated amount of urolithin A in each of the five Sardinian pig bone samples (NF-not found) (mean values n = 5). (**d**) A list of percentage binding to hydroxyapatite (HAP), for polyphenolic compounds chosen to demonstrate a range of molecular weights and structures. Further details along with compound structures provided in an expanded table in SI (Supplementary Fig. 6 and Table 3).

In order to identify plant-derived compounds in the bone extracts we compared accurate mass, chromatographic retention time, isotope patterns, and fragmentation patterns for each compound feature (with a signal to noise ratio of at least 3:1) with the same measurements for the authentic standards. Five plant-derived compounds matched extremely closely: Urolithin A, urolithin B, quinic acid, *p*-coumaric acid, and elaidic acid (accurate mass (<3 ppm), retention time (<0.01 min), isotope patterns (>97%) and product ion spectrum (CID) matching at least the base peak and two additional product ions). Figure 1(c–f) illustrates these data for the identification of urolithin A from the analysis of Sardinian pig bone 6070116). Figures 2b and 3a provide details of the relative abundances and matching of authentic standard measurements with those of sample extracts. Supplementary Figs 2–5 compare the product ion spectra for each of the identified plant-derived compounds between authentic standards and the sample extracts. These data illustrate that the major peaks in the product ion spectra match those of the authentic standard providing additional confirmation of structural similarity (small differences in matching of minor peaks are likely to be due to losses resulting from the low intensity of sample precursor ions compared to those of the standard). Elaidic acid was identified in all 5 Sardinian samples and Iberico bone extracts but not in extracts from the negative control. *p*-Coumaric acid was identified in all 5 Sardinian samples but not the Iberico samples or in the negative control. Quinic acid was identified in all Sardinian samples and Iberico samples and was also found in the negative control (Supplementary Fig. 1c). All 5 compounds were present in the acorn supplement (Fig. 1a,b). Although Urolithin A & B, quinic acid and *p*-coumaric acid are not known in endogenous metabolism of vertebrates, elaidic acid is an endogenous metabolite according to the Human Metabolome database (HMDB). It is therefore unclear whether the levels of elaidic acid measured in the bone extract reflect endogenous or exogenous sources for this particular metabolite despite it not being observed in the negative control for the acorn diet.

Of note was the absence of the ellagitannins (Urolithin A & B) in the bones of one of the Sardinian ‘growers’ (6070125) (Fig. 3a). We do not have a conclusive explanation for this false negative result but this particular pig was known to have suffered from poor feeding performance, weight gain and feed conversion rate compared to the other pigs. In general, weight losses were observed in growers immediately after weaning with a subsequent compensatory growth, but this pig in particular displayed the worst average daily gain. It is interesting to speculate whether the microbiome played a role here as it is well documented that urolithins are produced from ellagic acid via microbial transformation in the gut prior to absorption. None of the pigs were given antibiotics or other dietary supplements however microbiome dysbiosis (potentially brought on by malnutrition, parasites or other infection) could explain both lower feeding performance and weight gain as well as the lack of urolithin formation. Whether caused by the unusual diet or not, it highlights the potential for false negatives and potential challenges for dietary reconstruction.

Figure 2a illustrates the relative abundances of urolithin A and urolithin B in the bone extracts from the 5 acorn fed Sardinian pigs and controls. This shows a differential distribution correlating with the two different pig rearing regimes (see ‘finishers’ and ‘growers’ in the Experimental section). The two ‘finisher’ pigs, (1196353 & 1196497), which were 21 weeks old at the time they underwent the 4-week feeding regime, show markedly higher levels of urolithin B and lower levels of urolithin A compared to two of the ‘growers’ (6070116 & 6070117) which showed the opposite trend with higher urolithin A and lower urolithin B (no Urolithin A or B was identified in 6070125). At least two factors may be significant: (i) there was a difference in age at which the two ‘growers’ and ‘finishers’ began the feeing regime (4–5 weeks for the ‘growers’ and 20–21 weeks for the ‘finishers’), and (ii) unlike the ‘finishers’ the ‘growers’ underwent a 15 week ‘wash out’ period feeding on a conventional diet (without acorn supplement) 4 weeks of acorn supplementation, before slaughter. An external calibration curve was created for Urolithin A (Fig. 2d) and the absolute amount of urolithin A in the extracts from each of the 5 bone extracts was calculated. It was found to be between 0.75 and 3.1 ng of Urolithin A per gram of bone (Fig. 3c).

## Evidence for the Accumulation of Urolithins in ‘Bone’ but not Associated ‘Fat’ or ‘Flesh’

The modern pig bones used in this study showed surface traces of both adipose tissue and in some cases muscle tissue. Care was taken to sample only ‘bone’ during the experiments, but is was possible that the polyphenols identified were derived from the fat and flesh traces associated with the bone samples, rather than the hard bone itself. In order to differentiate between these possible sources, adipose and muscle were isolated separately from the surface of the same piece of bone, from the acorn-diet fed Sardinian pig (6070116). The same extraction protocol was then used separately for the adipose tissue, muscle tissue and bone using the same weight of each (2 grams). No evidence was obtained for the presence of urolithin A, urolithin B (or other ellagitannins) in any of the adipose or muscle extracts but both were identified in the bone extract as expected (data not shown). These results suggest that diet-derived urolithins were present in hard bone but not associated soft tissues at levels that could be detected.

## The Selectivity of an Untargeted Approach to Plant-Derived Biomarker Discovery

Principle components analysis (PCA) was performed on data from the analysis of Sardinian and control pig bones, using all compound features. Figure 3b shows the scores plot where samples from pigs fed acorn supplemented diets cluster together, but separately from the positive and negative controls. This indicates that the solvent extraction process was both reproducible and sensitive to differences in metabolite composition.

The identification of plant-derived polyphenols in the Sardinian bones, described in the section above (*Diet-derived plant polyphenols are found in animal bones),* was determined by comparison with authentic standards which were chosen based on the compound composition of the acorn supplement. Next we investigated how selective this approach might be without prior knowledge of dietary composition. We used the human metabolome database (HMDB) which contains over 75,000 compounds, many of which are plant-derived^19^. We searched our dataset (>26,000 compound features as represented by the PCA plot in Figure 3b) against the full HMDB database using a 3 ppm accurate mass cut-off. Five thousand eight hundred and fifty three putative identifications based on accurate mass were returned. Of these 22 putative matches were made to the 5 compounds already identified (urolithin, A, urolithin B, elaidic acid, quinic acid and p-coumaric acid). When an isotope similarity score of >90% was applied as an additional identification criterion (a theoretical value), this reduced the matches to 16. When a retention time window of 0.5 mins (compared to the authentic standard) was introduced, a single match for urolithin A, urolithin B, quinic acid and p-coumaric acid was achieved. Elaidic acid retained 2 possible candidates down to a retention time window +/−0.1 mins. Given that it is a lipid with at least 10 endogenous structural isomers known, this is perhaps not surprising. In general, the precision and accuracy of polyphenol identification obtained when chromatographic retention time was included as a discriminating factor, showed that a database search, followed by use of authentic standard retention times, was an appropriate approach for obtaining accurate identifications. This demonstrates the potential for using an untargeted, discovery-led method to identify plant-specific polyphenolic compounds in bones without prior knowledge of dietary composition.

## A Wide Range of Polyphenols Bind Strongly to Hydroxyapatite

In order further investigate the binding of plant-derived polyphenols to bone we developed and tested a model in which the affinity of a range of polyphenols for hydroxyapatite, the mineral component of bone, was explored^20^. We mixed polyphenol standards with HAP and established the degree to which they bound (see experimental section for details). We found a wide range of affinities from 0 to 100% of the polyphenol in solution binding to HAP. Figure 3d shows a selection of these compounds and their percentage binding. The full list of compounds tested, along with additional analytical information, can be found in Supplementary Table 3 and Figure 6 provides the structures of each of these compounds. Examining the chemical structure of compounds which bound strongly showed binding was associated with the presence of three factors: (i) the presence of multiple hydroxyl groups on the polyphenol (ii) adjacent hydroxyl groups or adjacent hydroxyl and carbonyl groups, and (iii) the presence of a carboxylic acid group in the molecule. Polyphenols that bound relatively weakly to HAP did often contain hydroxyl and carbonyl groups, but they tended not to be adjacent and lacked a carboxyl acid group in addition.

This simple model did not aim to reproduce physiological conditions, hence it is not suggested relative binding effects reported here will necessarily reflect those *in vivo*. The primary aim was to see whether binding of different types of polyphenol took place and, if so, whether this could be linked to chemical structure, which is what we found.

## Discussion

In the plant kingdom most polyphenols occur in their glycosylated forms^1^. 5–10% of the total polyphenol intake, may be directly absorbed in the human small intestine as the aglycone form after deglycosylation, the rest is subject to de-glycosylation, microbial metabolism and abortion of these metabolic products in the colon. Most polyphenolic components are subsequently bio-transformed (mainly methylated, sulphated or glucoronated) before entering circulation^4^. However, some polyphenols (for example certain flavones, isoflavones, catechins and hydroxycinnamic acids), have been shown to be present as the aglycone in circulation at levels of a few percent to over 20%^21^.

Ellagic acid is known to be extensively metabolised by pig and human gut microbiota to form a range of urolithins of which urolithin A and urolithin B are particularly prevalent^22,23^. Both have been identified at elevated levels in human blood plasma and urine after ingestion of ellagic acid^24^. This helps explain the absence of ellagic acid in the pig bone extracts in this study but relatively high levels of urolithin A and B. Iberian pigs have also previously been used as a model for studying urolithin A production from dietary ellagitannins and, although urolithin A in its free form was found in urine, bladder, and gall bladder tissue, it was not found in other tissues studied including muscle, adipose tissue, liver, kidney, and heart^16,25^. We can find no evidence of bone having previously been analysed for the presence of ellagitannins.

It might be expected that the un-conjugated (aglycone) form of polyphenols would bind most effectively with calcium in bone as conjugation generally removes the more polar hydroxyl groups expected to form a coordination complex with hydroxyapatite-derived calcium ions. Indeed the polyphenols we identified in the bone extracts were not conjugated, however the levels of un-conjugated polyphenols in plasma is reported as low or absent for most polyphenols studied by others. This raises the question of whether the un-conjugated polyphenols we identify in bone came directly from the low abundance, un-conjugated pool in plasma or were previously conjugated in circulation (a much larger pool) and underwent de-conjugation before sequestration into bone. These questions are of relevance with respect to potential applications in dietary reconstruction for example as the two pools are likely to represent different polyphenolic profiles. Further investigation is needed but there are a number of possible mechanisms available for de-conjugation of polyphenols in plasma and in cells including osteons. It is known for example that at least some types of conjugated polyphenol can enter cells via passive and active transport having been de-conjugated outside the cell via glucuronidase and other de-conjugating enzymes^21^. Intracellular de-conjugation of glucuronides is also known to increase during inflammation and it has been shown that when polyphenols are present at high levels in plasma, saturation of conjugation pathways can lead to increased presence of aglycones in circulation^21^.

Another intriguing possibility is that circulating polyphenols may be de-conjugated at sites of bone growth specifically in the osteoblastic environment. Osteoblasts are located in highly vascularised osteons close to a ‘cement line’ where mineralisation of the organic bone matrix takes place^8^. Osteoblasts have extensive capacity for removing protons, water and transport of phosphates and calcium ions in order to facilitate bone formation; this environment has a considerably raised pH^8^. When calcium and phosphate are transported across secretory osteoblasts, it has been shown that calcium-binding fluorescent molecules (including tetracycline) are co-transported^26^. Calcium binding fluorescent dyes and phosphate analogues (bisphosphonates) have been shown to also bind to the mineral matrix during bone formation^27^. We speculate that an analogous mechanism transports a range of plant-derived polyphenols, such as the urolithins, to the calcium-rich (high pH) region of mineralisation. Tetracycline is a relatively large, unconjugated compound with multiple hydroxyl groups, similar structurally to polyphenols, suggesting compounds of similar size or smaller may be transportable to sites of mineralisation (Supplementary Figure 6/No.89). Conjugated polyphenols are chemically less stable at higher pH and the alkaline conditions found in osteons may be sufficient to promote chemical de-conjugation if conjugated polyphenols are in fact transported there from circulation.

In this paper we show evidence that urolithins, derived from microbial breakdown of ellagitannins in the gut, as well as additional plant-derived polyphenols, are sequestered into the bones of pigs from the diet and accumulate to levels elevated above surrounding tissues. Multiple possible absorption mechanisms, combined with microbial metabolism and conjugation, mean that the polyphenol profiles associated with bone may not reflect consumption profiles directly. The mechanisms of sequestration into bone may also bias towards compounds which form strong complexes with calcium and/or polyphenols which are absorbed as aglycones or de-conjugated most efficiently in the local environment of the osteon. This study provides evidence that a range of plant-derived biomarkers of dietary consumption can be found in bone, and introduces a methodology for their isolation and identification. Plant polyphenols can be specific to certain species of plant and plant-derived food categories. Considering there are currently virtually no methods that provide evidence for the consumption of specific plant products from the analysis of bone, the approach described here provides potential for applications in forensics and palaeodietary reconstruction. Studies to investigate the preservation of such molecules in bone subject to depositional environments, are required to demonstrate this potential further.

It has not escaped our attention that some of the polyphenols we identified in bone can have specific positive health effects including recent studies showing that urolithin A has anti-proliferative and anti-cancer activity in prostate and colon cancer cells^28,29^ and that other polyphenols, and polyphenol-containing diets, can positively affect bone growth with respect to osteoporosis^30^. Mechanisms remain unclear but appear to go beyond general anti-oxidative and anti-inflammatory responses, with polyphenols influencing osteoblast and osteoclast differentiation directly in osteoporosis, by an as yet unknown mechanism^31^. Whether dietary urolithins and other polyphenols provide prophylactic, therapeutic or other health effects, or can modulate bone growth and/or re-adsorption directly, remains to be further investigated.

## Materials and Methods

### Experimental design

The objectives of this study were to (1) to rear domesticated pigs on a diet rich in acorns. (2) Identify the polyphenolic composition of their feed at the individual compound level. (3) Look for evidence of corresponding polyphenols in extracts from the bones of these animals post-mortem to provide evidence for the sequestration of dietary polyphenol in growing bone. (4) Look for evidence of interactions between polyphenols and hydroxyapatite which might help explain the associations found in bone.

A total of 5 cross-bred pigs (Duroc sires on Large White X German Landrace, non-acclimated to acorns), consisting of 3 ‘growers’ (initial body weight, BW: 7.15 to 7.65 kg; age: 4 to 5 weeks) and of 2 ‘finishers’ (initial body weight, BW: 113 to 115 kg; age; 20 to 21 weeks) were involved in the same feeding trial. After an adaptation period of 10 days when all animals were fed the same pelleted complete diet (phase 1), all pigs were switched to the experimental diet which was composed of 50% of the same pelleted complete diet while the remaining 50% composed of ripe, hulled and shredded acorns, fed in a mixed meal form. The experimental diet was formulated to test the effect of one single ingredient (acorn), combined with a reference diet in known proportions. Both in growing and finishing pigs were given the same experimental diet and the experimental feeding lasted four weeks for all pigs. The composition of the experimental diet is reported in Supplementary Table 4. Individual feed intake was recorded daily, by subtraction of leftovers weighed in the feeder from the offered feed the day before, split in two administrations over 24 h. On the basis of weight and chemical composition of feed offer and leftovers, daily intakes of dry matter (DM) and tannic acid equivalent (TAE) in feed were calculated per kg BW for each pig. The pigs were split into two categories comprising three ‘growers’ and two ‘finishers’. The ‘growers’ and ‘finishers’ differed in the age at the start of the diet administration and the length of time between completion of the experimental dietary regime and date of slaughter. However both groups were exposed to the same experimental acorn diet for the same period of time (4 weeks).

All of the pigs (growers and finishers) appeared healthy throughout the feeding trial except for the slow growth of 6070125 reported in the manuscript. Daily intakes of feed (on DM basis) and consequently of TAE, varied between growers and finishers. In younger pigs the intake of DM was proportionally higher (when referred to DM capacity in relation to live weight) according to the growth stage. This was to such an extent, that the higher the TAE intake the lower the body mass gain per day resulted^32^. This datum is interpreted as a depressive effect of appetite due to tannin rich diet, as observed in several animal species^33^. This means that proportionally, growers ingested higher TAE amounts per kg BM per day than finishers did. Intakes and yields (final BM) are reported in Supplementary Table 5. Growers continued to be raised until the age of 25 weeks and no acorns were supplied with the conventional diet. This meant that growers underwent a ‘wash out’ period prior to slaughter. Finishers were slaughtered on day 29.

### Metabolite standards and their preparation

Urolithin A, B and C were purchased from Dalton Pharma Services (Dalton Pharma Services, Toronto, Canada). All other standard compounds, including all solvents, were purchased from Sigma-Aldrich (Gillingham, Dorset, UK). All solvents used were HPLC grade or above where applicable. Analytical solutions, of all the standards used, were prepared at 5 µg/mL unless otherwise stated and made up in 80% EtOH or 80% MeOH using HPLC grade solvents and de-ionised, Milli-Q water (Millipore, Burlington MA, USA).

### Animals and ethics statement

All animal handling, protocols and methods complied with the recommendation of the European Union Directive 86/609/EEC and Italian law 116/92 concerning animal care.

Experimental procedures were approved by the Ethics Committee of Hannover district and replicates carried out on a commercial farm following EU Directive 86/609/EEC and Italian Law116/92, in respect of the three Rs principal (Reduction, Replacement and Refinement) associated with animal experimentation. The TAE amount in diet was calculated to meet EFSA safe limit for animal health, public health and environmental protection. Supplementary Table 1 provides details of all modern bone samples and their provenance used in this study.

### Chemical analysis of feed

Chemical composition of the diet was determined by Weende analysis described by Naumann and Bassler^34^. Polyphenols showing a protein precipitating activity at polyvinyl-pyrrolidone adsorption were estimated using the Folin-Ciocalteau^35^ method, modified by Waterman and Mole^36^. Contents were expressed as g of Tannic Acid Equivalents per kg dry mass in the diet^35,36^.

### Untargeted metabolite analysis by mass spectrometry (LC-MS/MS)

LC-MS/MS analyses were performed using two different LC-MS/MS systems. The first was a Thermo Scientific U3000 ion chromatography system coupled directly to a Q-Exactive HF Hybrid Quadrupole-Orbitrap mass spectrometer with a HESI II electrospray ionisation source (Thermo Scientific, San Jose, CA). The U3000 HPLC system incorporated binary pumping system, autosampler with 25 µL sample loop and 25 µL syringe. A heated column manager was maintained at 40 °C. Details of the LC-MS/MS method used for untargeted metabolic profiling, was previously published^37^. We briefly describe the method again here: A 25 μL partial loop injection was used for all analyses and the chromatographic separation was performed using a Waters Acquity HSS T3 UPLC column (2.1 × 100 mm, 1.8 uM), without a guard column. The chromatographic flow rate was 0.3 mL/min and the total run time was 18 minutes. Mobile phase A consisted of 0.1% formic acid in Milli Q water and mobile phase B: 0.1% formic acid in MeOH. The gradient used for elution comprised as follows: 0 min, 5% B; 4 mins, 50% B; 12 mins, 99.9% B; 15 mins, 99.9% B; 15.1 mins, 5% B; 18 mins, 5% B. Mass spectrometry was performed in negative ion mode using a scan range from m/z 60–1000 and the resolution was set to 70,000. The tune file and source parameters were: Sheath gas flow 25; Aux gas flow 8; Spray voltage 3.5; Capillary temperature 320; S-lens RF value 70; Heater temperature 300. AGC target was set to 1e6 and the Max IT value was 250 ms. Full scan data were acquired in continuum mode and combined with data-directed MS2 scanning to provide data directed fragmentation spectra at 17,500 resolution. The MS/MS settings were as follows: AGC 1e5, Max IT 50 ms, loop count 5, isolation window 1.0 m/z, NCE 30, intensity threshold 2e4.

LC-MS/MS analysis was also performed for confirmation purposes using an Agilent 6550 Accurate-Mass Quadrupole Time-of-Flight mass spectrometer coupled to an Agilent 1290 UHPLC systems (Agilent Technologies, Santa Clara, CA, USA). The chromatographic method used was the same as detailed above. Parameters for MS analysis were similar as above except for: Sheath gas flow 12; Max IT value 333.3 ms.

MS/MS settings on the Agilent system were as follows: Acquisition rate 2 spectra/s, acquisition time 500 ms/spectrum, isolation window 1.3 Da, intensity threshold 2e4. Targeted m/z values were: 191.055, fragmentation energy 10 V; 227.034, fragmentation energy 30 V; 211.039, fragmentation energy 30 V; 300.998, fragmentation energy 30 V.

### Processing of LC-MS/MS data

Raw data was processed using Xcaliber software version 2.2 (Thermo Fisher Scientific, Waltham, MA, USA) for data obtained from the Q-Exactive and Masshunter for Agilent Data (Agilent Santa Clara, CA, USA). Progenesis QI for small molecules (Waters, Elstree, UK) was used for measuring the number of compound features (compound features had a unique m/z/chromatographic retention time and isotope peaks corresponding to their expected natural abundances). Compound identification and statistical analysis was also performed using Progenesis QI. The Progenesis workflow involved automatic peak alignment, isotope cluster recognition (‘peak picking’), compound identification using a database constructed from the authentic standard analysis (see below) and statistical analysis including PCA analysis. This included the comparison of normalised molecular abundance across samples for identified and unknown compounds. The relationship between peak area and concentration, for a particular compound feature, was used to compare concentrations between samples, when above the limit of quantification (LOQ). The LOQ was determined by ensuring it was above the instrument detection limit where applicable (urolithin A) and a signal to noise ratio of 10:1 (see experimental section below).

### Determining limit of detection (LOD) and limit of quantification (LOQ)

The limit of detection (LOD) and limit of quantification (LOQ) for LC-MS/MS experiments is often determined by identifying a signal to noise ratio measurement of 3:1 and 10:1 respectively. Here we performed a similar experiment by spiking the sample matrices (control pig bone extract) with different concentrations of urolithin A in order to produce calibration curves for each standard and use these to establish the concentration at a signal to noise ration 1:3 and 1:10 corresponding to LOD and LOQ respectively. The signal was determined as the maximum height of the extracted ion chromatogram peak for each analyte and the noise was determined as the average of the maximum and minimum intensity of the signal 60 seconds before and 30 seconds after the analyte peak.

It has been argued that relying on a S/N measurements can be too simplistic for determining the limit of quantification as it does not capture information about analytical variability associated with repeated analyses and is subject to vendor/instrument differences in algorithms used to reduce background noise levels. An alternative approach is to calculate an instrument detection limit (IDL) which is not based on an absolute background level measurement but instead uses a statistical measure of repeated analyses of the sample at a concentration close to the limit of detection alongside repeated analysis of blank samples. The average blank value is subtracted from each of the analyte measurements to remove any background contribution and the use of multiple measurements enables a statistical significance (and therefore probability level) to be determined which captures the sample-sample variability. The IDL is the amount of sample that provides a signal that is statistically greater than the population mean value of zero. This is determined from the following equation.

$${\rm{IDL}}={\rm{t}}\,\times {\rm{SD}}\,\times \,{\rm{amount}}\,{\rm{measured}}$$

where t = the statistical test from a one sided student t-test distribution and SD = standard deviation of the lowest concentration of the analyte that allows a statistical difference to be determined.

To determine the instrument detection limit on the U3000 coupled to the Q-Exctive MS systems we created an initial external calibration curve to estimate the concentration close to the limit of detection. This was 8 pg/mL. The average peak area at this concentration was 4878 (10 replicate analyses) and the standard deviation was 1670. The T-value at 99% was 2.821. We therefore were able to calculate the instrument detection limit as 9.03 pg/mL with a 99% confidence limit. The instrument detection limit was not determined for the Agilent LC-MS system applied for confirmatory analysis, however all spectral identifications had a signal to noise ratio >10:1.m

### Metabolite identification

Identification of compounds in experimental samples was based on matching of up to four orthogonal parameters via an in-house library constructed from measurement of authentic standards. The orthogonal parameters comprised: (1) accurate mass (<5 ppm) measurement compared to theoretical mass derived from the chemical formula, (2) experimental retention time derived from LC/MS analysis of authentic standards, (3) isotope pattern recognition based on experimental comparison with chemical formula prediction and (4) matching of MS/MS fragments with those of authentic standards. Further details of the compounds and authentic standards used are given in the manuscript and extended data sections.

### LC-MS/MS analysis of authentic compound standards

Individual polyphenol standards were diluted to 5 µg/ml using 100% EtOH. Each standard solution was analyses separately using the LC-MS/MS method described above which was also used for the analysis of acorn and bone extracts. The retention time, isotope abundance pattern and product ion spectra were obtained and used to construct a standard database that was searched for compound identification in the bone and acorn experimental samples.

### Extraction of compounds from acorns used in experimental diets

3 mL of 80:20 MeOH:H_2_O was added to 300 mg of acorn supplement powder in a 7 mL Hard Tissue homogenising tube (Precellys, Montigny-le-Bretanneux, France). The contents were homogenised for 9 cycles of 20 s at 4500 rpm with a 25 second pause. The mix was ultrasonicated at 50 Hz for 4 minutes in a Kerry Ultrasonic Bath. 1 mL of the upper part of the extract was transferred to a 1.5 mL ultracentrifugation tube and centrifuged for 5 mins at 13,000 rpm. The supernatant was filtered through a 0.45 µm nylon syringe filter and collected in an autosampler vial for analysis.

### Preparation of bone samples

De-fleshing and removal of bone marrow from each femur was completed mechanically. Bones were crushed and stored in individual tubes prior to shipment to the UK where they were stored at −80 until the day of sample extraction.

### Extraction of compounds from bones

In order to extract plant-derived compounds from bone samples a number of approaches were investigated. It was expected that compounds of interest may be at very low abundances relative to other endogenous compounds such as lipids, proteins and inorganic ions. The following methodological approaches were investigated: (1) direct solvent extraction which included homogenisation and sonication methods. (2) Supercritical fluid extraction (SFE) and 3) Soxhlet solvent extraction. Direct solvent extraction with prior sample homogenisation, emerged as the most efficient and reproducible method (data not shown) for extraction of small molecules from bone material. 2 g of bone was weighed into a 15-mL homogeniser tube (Precellys, Montigny-le-Bretonneux, France) which contained 4 ceramic (zirconium oxide) 6.8 mm beads. Homogenisation took place using the following settings: 8 × 20 s at 6200 rpm with a 25 second pause. 8 ml of 1% formic acid in EtOH was added to the homogenate. The homogenization protocol was then repeated as follows: 6 × 20 s at 5000 rpm with 25 second pause. Once complete the liquid homogenate including beads was transferred into a 15 mL falcon tube, left overnight at 4 °C and centrifuged at 4500 rpm for 30 mins. The supernatant was then carefully removed and filtered through a 0.45 µm nylon filter. The supernatant was diluted to a solvent ratio of 80:20 EtOH:H_2_O 500 µl supernatant was placed in a pre-rinsed 10 kDa molecular weight cut off filter and centrifuged at 14,000 rpm for 40 minutes. The resulting filtrate was filtered through a 0.45 µm nylon filter and transferred to an autosampler vial for analysis for direct LC-MS/MS analysis. A freeze-thaw cycle was avoided as it was shown this led to loss of polyphenolic compounds.

The following protocol was used for Supercritical Fluid Extraction (SFE). It is given here for completeness although it was not used for extractions from which results are reported. 2 g of bone was loaded into the extraction vessel of a JASCO supercritical fluid chromatography and extraction system (Jasco (UK) Ltd, Great Dunmow, Essex (UK)). The supercritical fluid extraction was achieved using CO_2_ mixed with an organic modifier (90:10 EtOH:H20 v/v). The proportion of modifier was fixed at 20%. The vessel was kept at 50 °C and 30 MPa during the extraction. Run times and flow rate were optimised to 120 mins and 3 mL/min respectively. The SFE extraction approach was effective for providing bone extracts based on recovery of known dietary biomarkers (see results). All data hereafter was derived from sample preparation using the liquid solvent extraction.

### Identification of polyphenols in bone solvent extracts

Untargeted LC-MS/MS analysis (as described above) was performed on the bone solvent extracts using the Thermo or Agilent LC-MS/MS system (depending on availability). Extracted ion chromatograms were applied for each targeted metabolite using a 5 ppm m/z cut-off. Where peak retention times obtained matched, or were very close to, the authentic standard retention time, and isotope abundances were within 90% of the theoretical value, a second targeted LC-MS/MS analysis was performed to collect product ion spectra and confirm metabolite identification. Such analyses were performed for ellagic acid, gallic acid, urolithin A, urolithin B and urolithin C in ethanol extracts from the Sardinian pig bones. Careful analysis of blanks and standards was used to check that the signals for the polyphenols, obtained from the analysis of bone metabolite extracts, were not the result of carry-over or contamination.

### Quantification of abundant compounds in acorn-rich diets

The absolute abundance of urolthin A in the Sardinian bone extracts was determined via generating an external calibration curve. A standard stock solution of urolithin A was made using 100% EtOH and diluted serially. The series of dilutions were analysed by LC-MS/MS and the extracted ion chromatogram peak areas for urolithin A were plotted against concentration. The curve for urothin A is shown in Fig. 2d. The equation for the line from the calibration curve was used to calculate the absolute concentration of urolithin A in the Sardinian pig bone extracts (Fig. 2d).

### Experiments to test the binding of polyphenols to hydroxyapatite (HAP)

Individual polyphenol standards were made up to a concentration of 5 µg/ml using 100% EtOH due to their generally poor solubility in aqueous solution. A 100 µL aliquot of the standard solution was place a total recovery vial for direct LC-MS analysis. 1 ml of the remaining solution was added to 75 mg of powdered HAP and mixed for 20 minutes. The mixture was then centrifuged at 4,500 rpm for 20 minutes at room temperature. The supernatant was filtered through a 0.45 µm nylon syringe filter and collected in a standard autosampler vial. Both samples (pre and post addition of HAP) were analysed consecutively using LC-MS. Extracted ion chromatograms were used to compare the peak areas of each polyphenol in both samples. The percentage loss (if any) of polyphenol from the solution post addition of HAP was calculated and this was used to determine the extent of binding to HAP. The assumption was made that any loss of polyphenol signal was the result of binding to HAP.

## Supplementary information

Supplementary Information

## Data Availability

The authors declare that the data supporting the findings of this study are available within the article and extended data sections. Any additional data not shown is also available upon request from james.mccullagh@chem.ox.ac.uk.
