# Supplementary material for: Metabolomics reveals diet-derived plant polyphenols accumulate in physiological bone
Source: Sci Rep. 2019 May 29;9:8047. doi: 10.1038/s41598-019-44390-1 (PMC6541599; doi:10.1038/s41598-019-44390-1)
Supplement: Supplementary file 1 — Supplementary Information [file 41598_2019_44390_MOESM1_ESM.pdf]

SUPPLEMENTARY INFORMATION

Metabolomics reveals diet-derived plant polyphenols accumulate in physiological bone

Isabelle Alldritt,[1] Beatrice Whitham-Agut,[1] Miguel Sipin,[1] Jacob Studholme,[1] Angela Trentacoste,[2] Jennifer A. Tripp,[3] Maria Grazia Cappai,[4] Peter Ditchfield,[5] Thibaut Devièse,[5] Robert E.M. Hedges,[1,5] and James S.O. McCullagh[1*]

[1]Department of Chemistry, Mansfield Road, University of Oxford, Oxford OX1 3TA, UK.
[2]Institute of Archaeology, University of Oxford, Oxford OX1 2PG, UK.
[3] UCL Institute of Archaeology, Gordon Square, London WC1H 0PY, UK.
[4]Research Unit of Animal Nutrition, Department of Veterinary Medicine, University of Sassari, Italy, [5]Research Laboratory for Archaeology and the History of Art, University of Oxford, Dyson Perrins Building, South Parks Road, Oxford OX1 3QY, UK.
*james.mccullagh@chem.ox.ac.uk

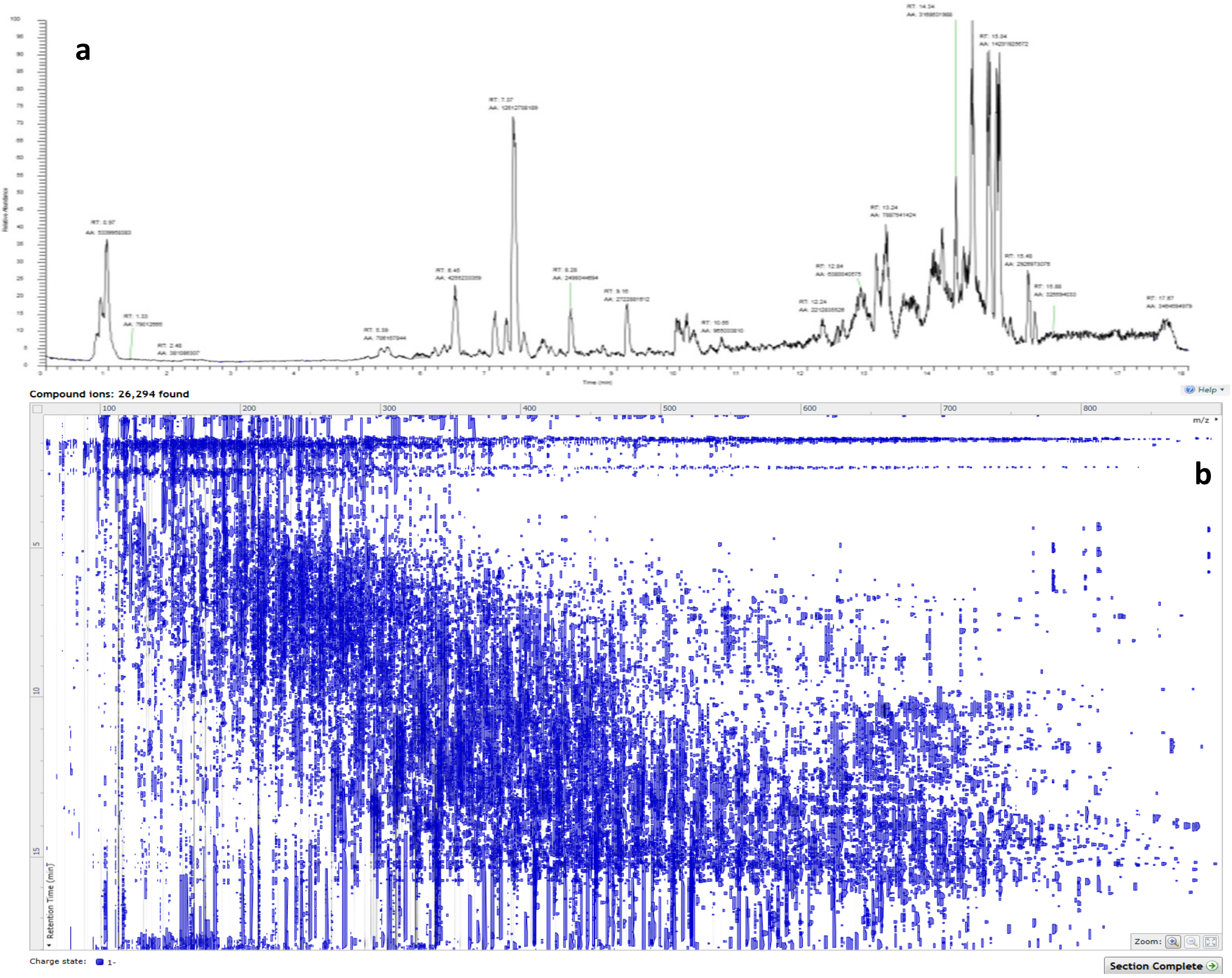

c

Sample	Urolithin A	Urolithin B	Elaidic acid	p-coumaric acid	Quinic acid
1196353	✓	✓	✓	✓	✓
1196497	✓	✓	✓	✓	✓
6070116	✓	✓	✓	✓	✓
6070117	✓	✓	✓	✓	✓
6070125	x	x	✓	✓	✓
Iberico	✓	✓	✓	x	✓
Market	x	x	x	x	✓

Supplementary Figure 1 | Plant-derived compounds identified in pig bones by untargeted metabolite profiling. **a,** Illustrative total ion chromatogram (TIC) for the untargeted LC-MS/MS analysis of Sardinian bone extract (60700116). **b**, Ion map from the untargeted LC-MS/MS analysis of Sardinian bone extract (60700116) shows distribution of over 26,000 compound features and their m/z values for untargeted analysis across the range *m/z* 60-1000. **c,** Plant-derived compounds identified in modern pig bone samples.

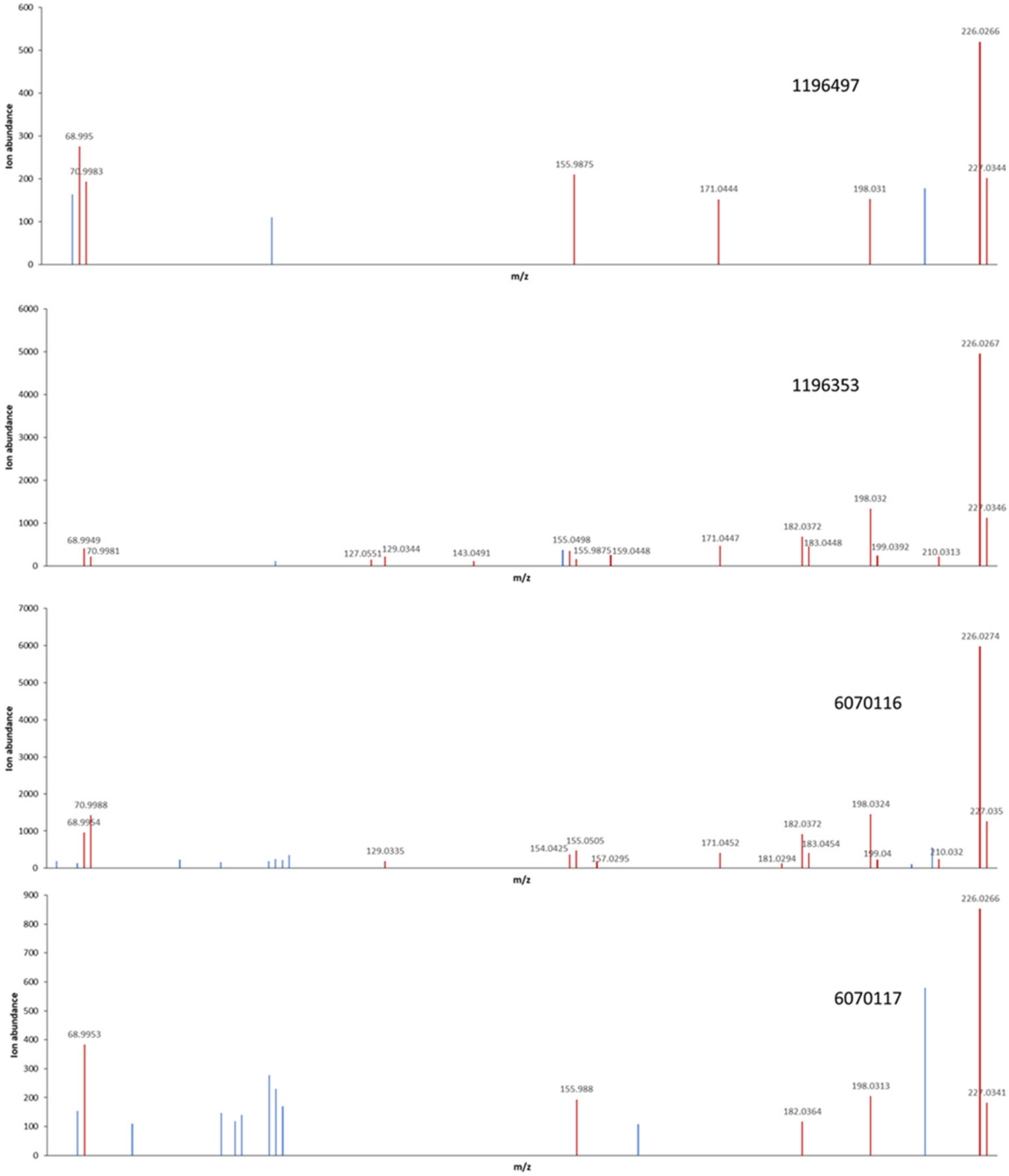

Supplementary Figure 2 | Product ion spectra for the CID fragmentation of the precursor for urolithin A acid in each Sardinian bone extract (no evidence of UA in sample 6070125 as discussed in the manuscript). Individual product ion m/z values matching those from the tandem mass spectra of the authentic urolithin A standard are shown in red (<7ppm).

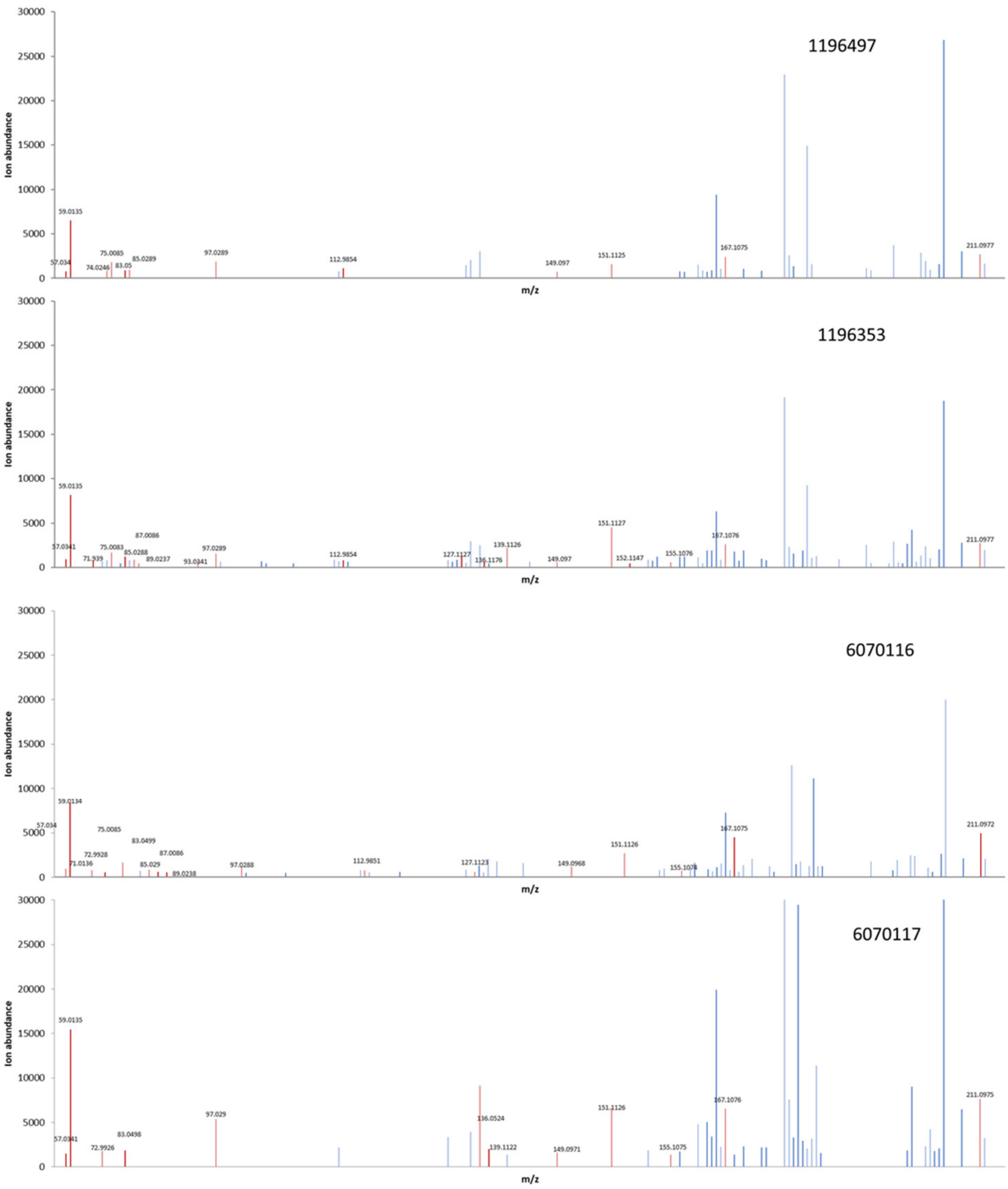

Supplementary Figure 3 | Product ion spectra for the CID fragmentation of the precursor for urolithin B acid in each Sardinian bone extract (no evidence of UB in sample 6070125 was found as discussed in the manuscript). Individual product ion m/z values matching those from the tandem mass spectra of the authentic urolithin B standard are shown in red (<12ppm). A significant number of matched peaks were identified although these represented a lower percentage than for urolithin A. The precursor ion m/z window was 1.5 dalton: Given the significant number of compound features (>18,000) some isobaric compounds ion or co-eluting species within the 1.5 dalton window, could lead to some ‘contamination’ of the product ion spectra. (<10ppm).

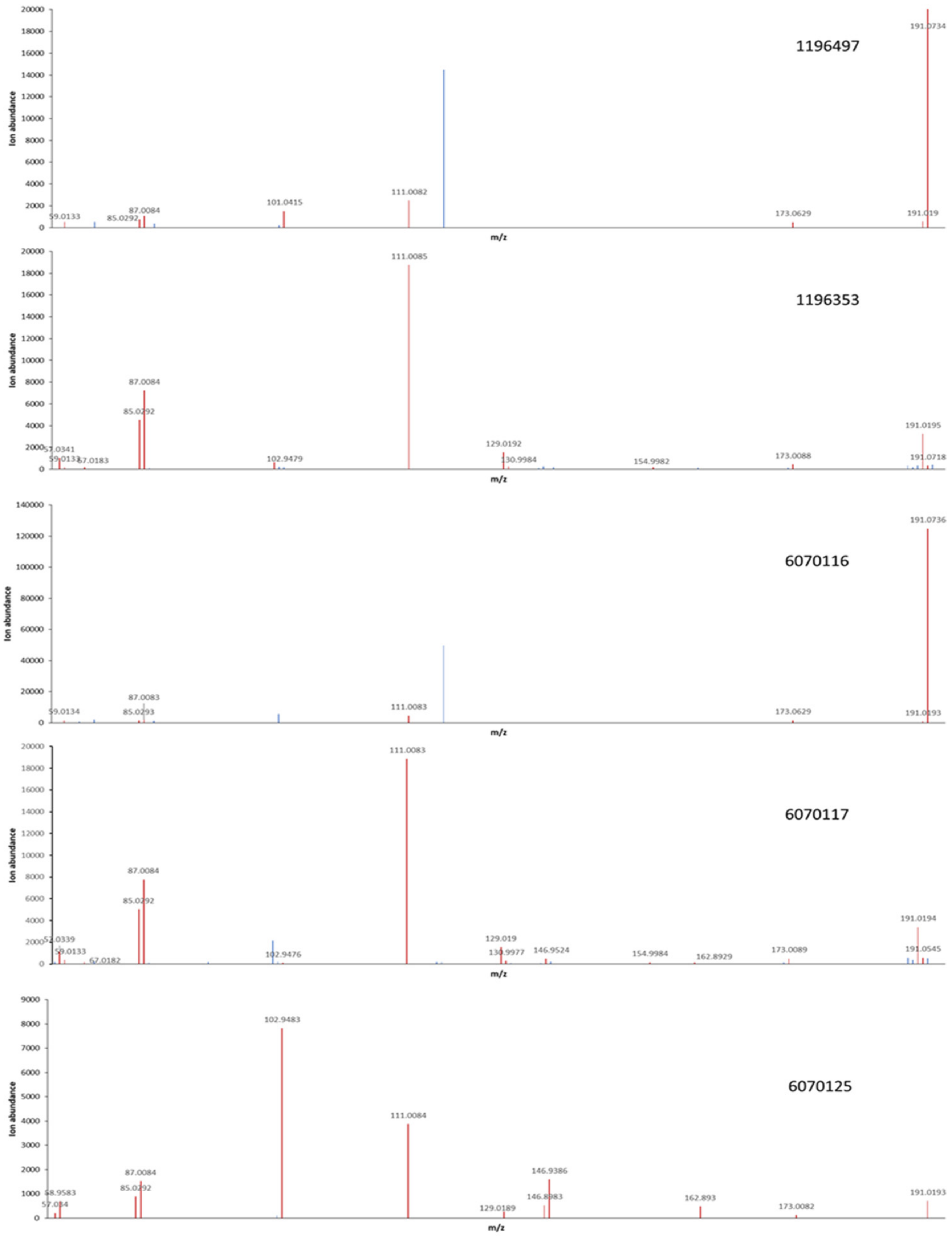

Supplementary Figure 4 | Product ion spectra for the CID fragmentation of the precursor for quinic acid in each Sardinian bone extract. Individual product ion m/z values matching those from the tandem mass spectra of the authentic quinic acid standard are shown in red (<8ppm).

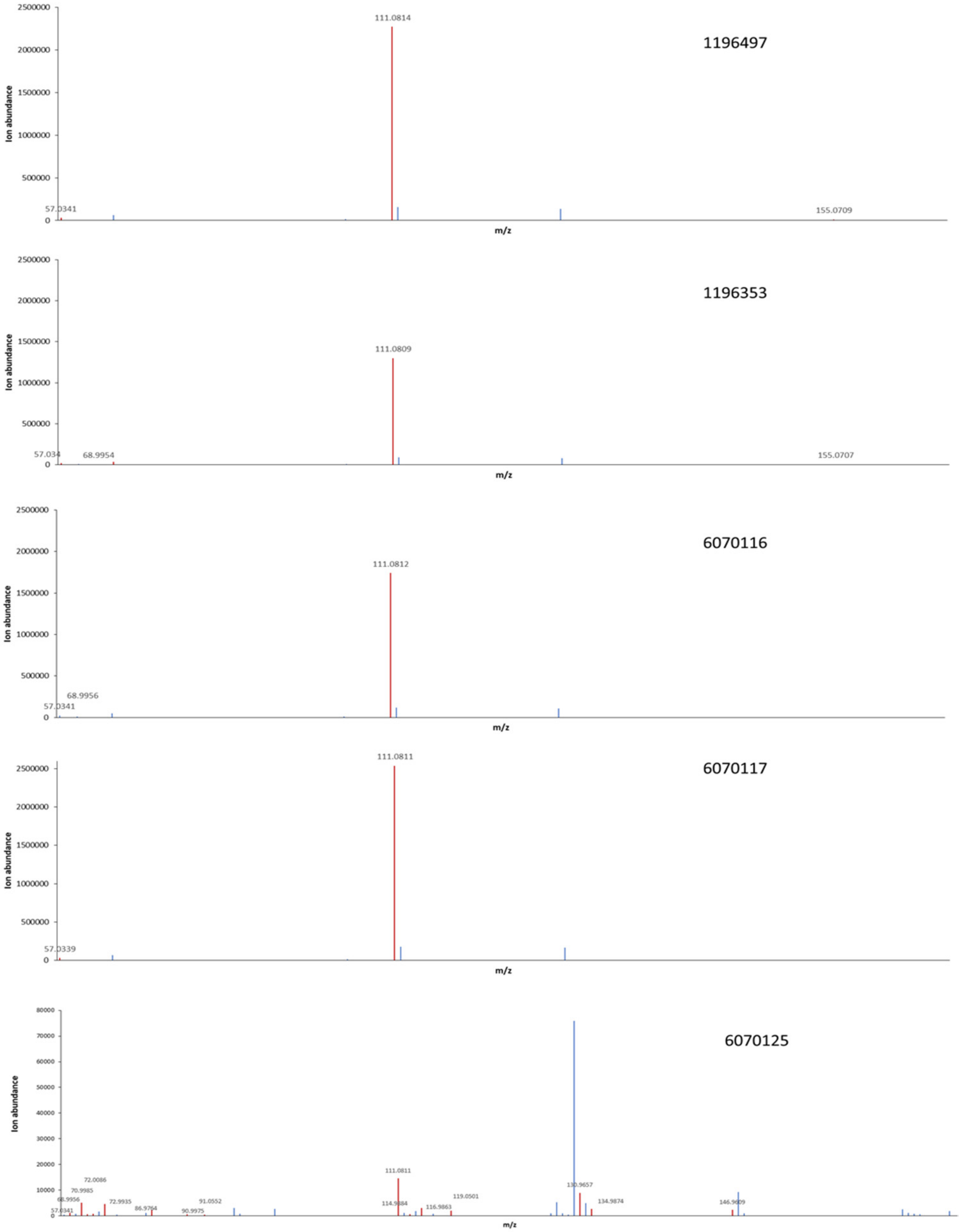

Supplementary Figure 5 | Product ion spectra for the CID fragmentation of the precursor for p-coumaric acid in each Sardinian bone extract. Individual product ion m/z values matching those from the tandem mass spectra of the authentic p-coumaric acid standard are shown in red (<6ppm).

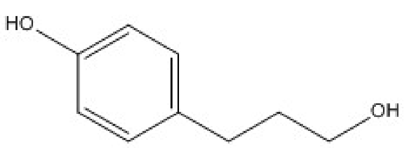

1 2-(4-hydroxyphenyl)ethanol

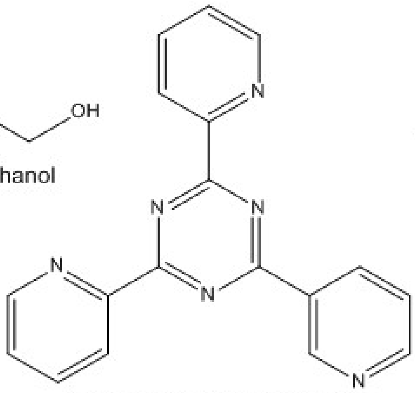

2,4,6-Tris(2-pyridyl)-s-triazine

2

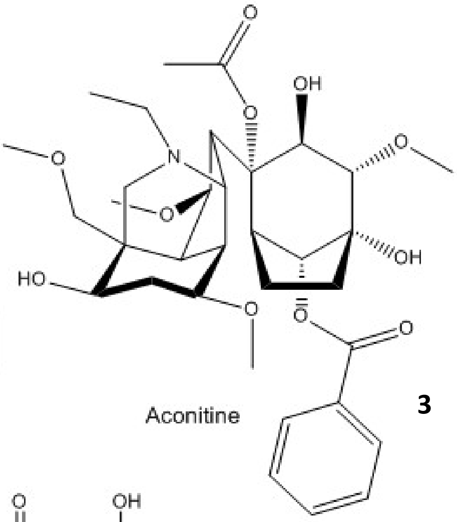

Aconitine **3**

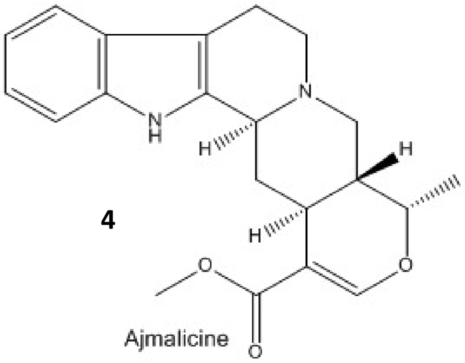

4

Ajmalicine

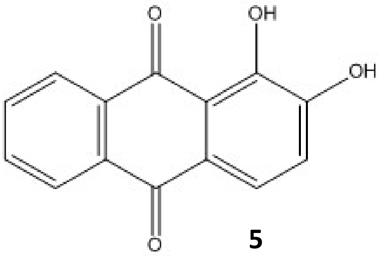

5

Alizarin

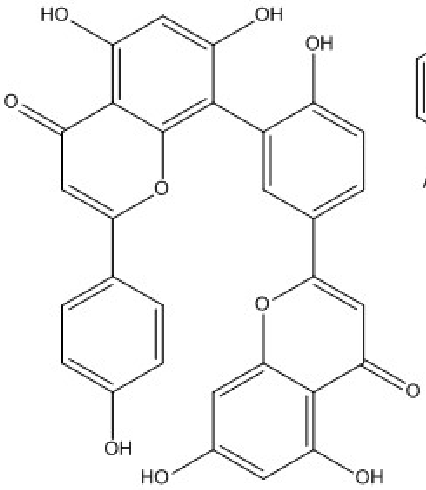

6 Amentoflavone

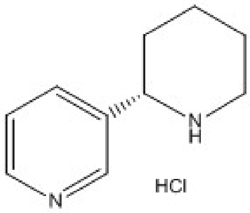

Anabasine hydrochloride

7

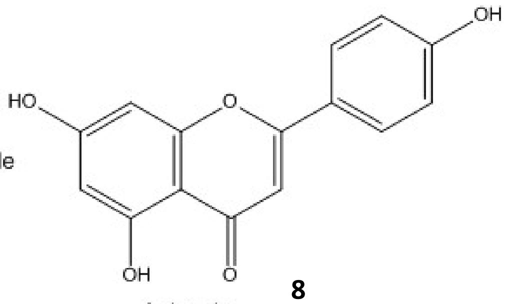

8

Apigenin

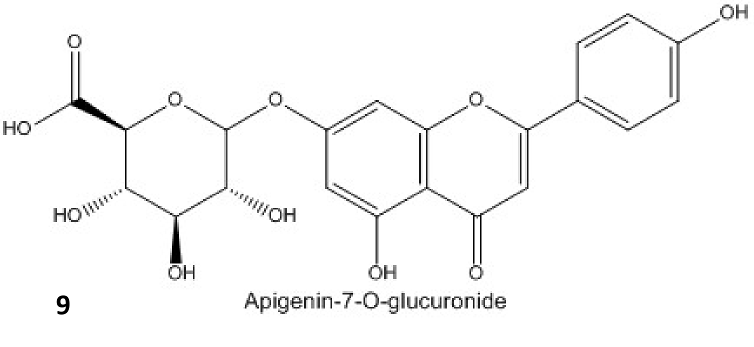

9 Apigenin-7-O-glucuronide

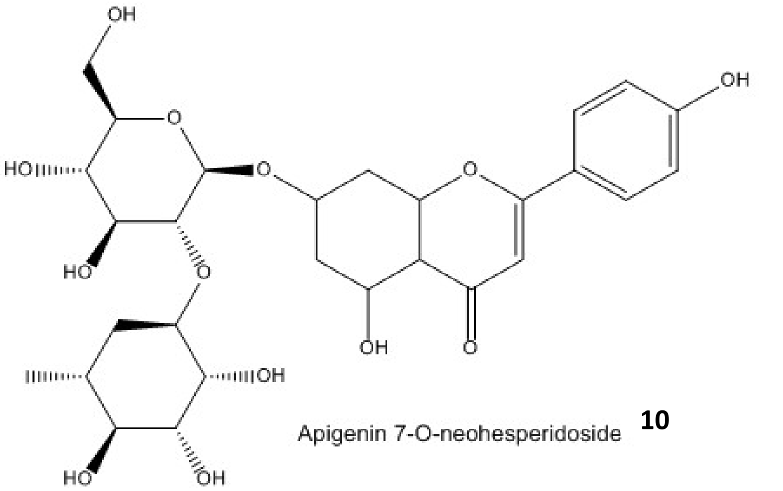

Apigenin 7-O-neohesperidoside **10**

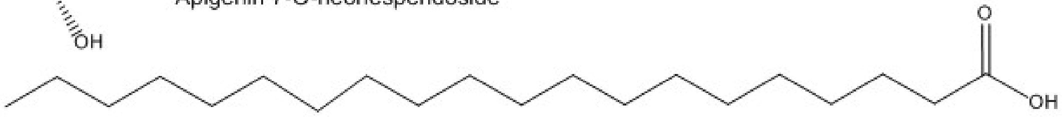

Arachidic acid **11**

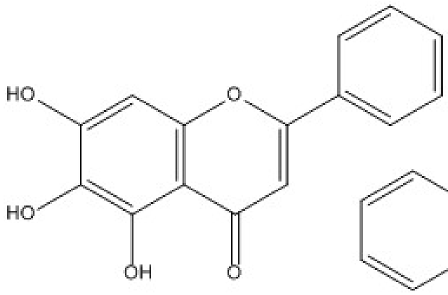

Baicalein

12

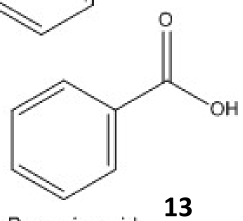

Benzoic acid **13**

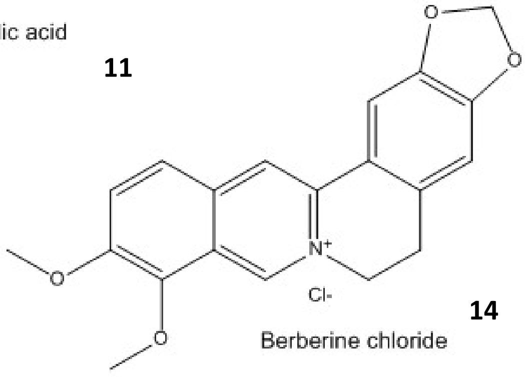

Berberine chloride **14**

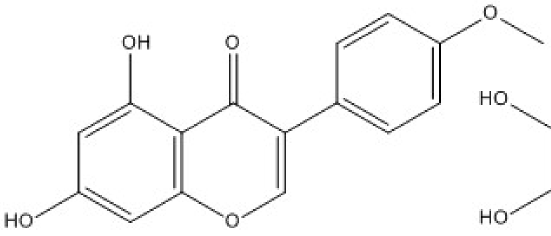

Biochainin A **15**

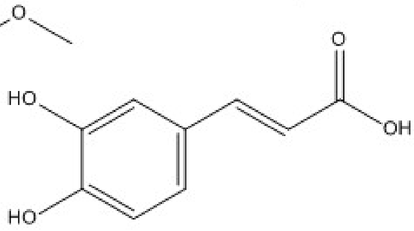

Caffeic acid

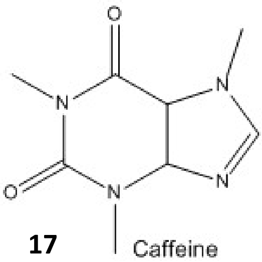

17 Caffeine

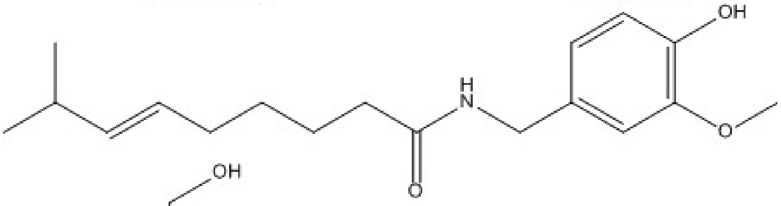

18

Capsaicin

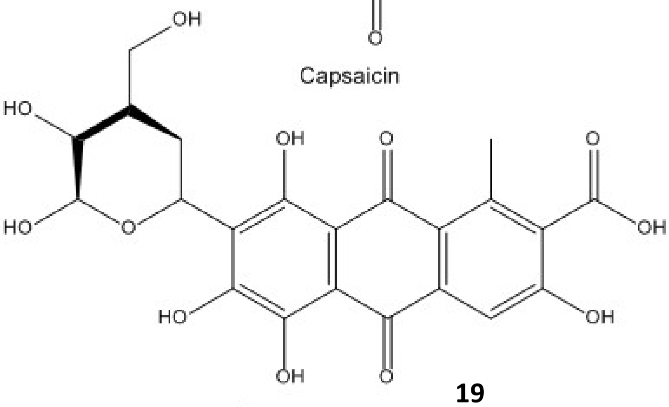

19

Carmine

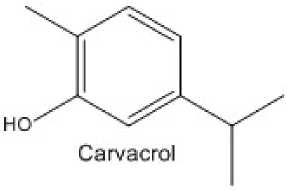

Carvacrol

20

21

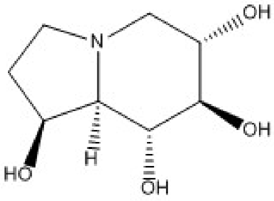

Castanospermine

22

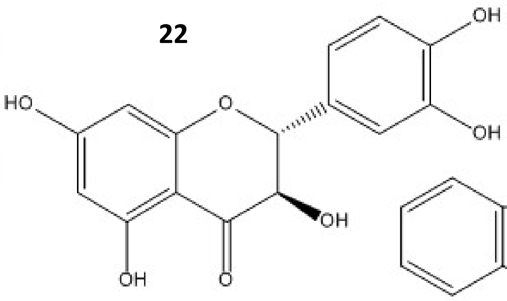

Catechin hydrate

23

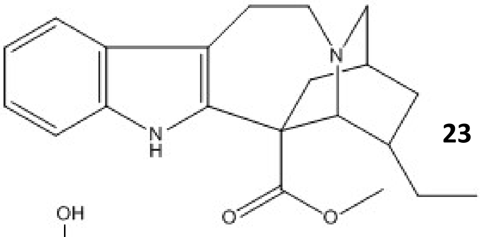

Catharanthine

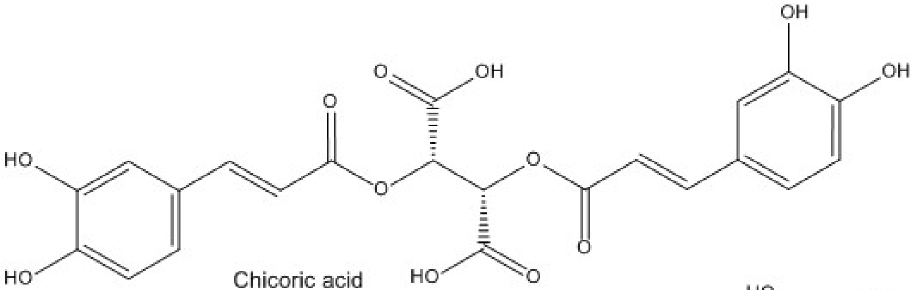

Chicoric acid

24

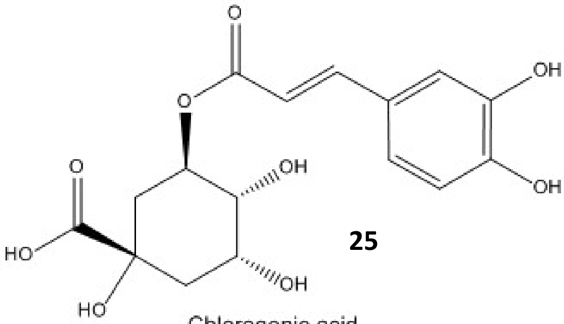

25

Chlorogenic acid

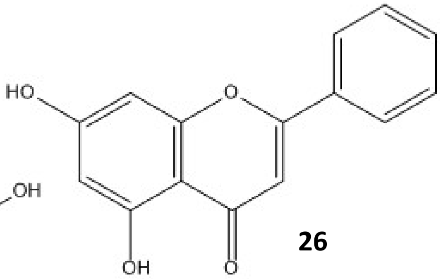

26

Chrysin

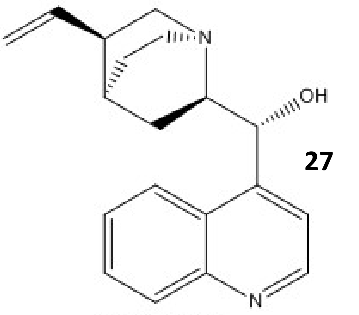

27

Cinchonidine

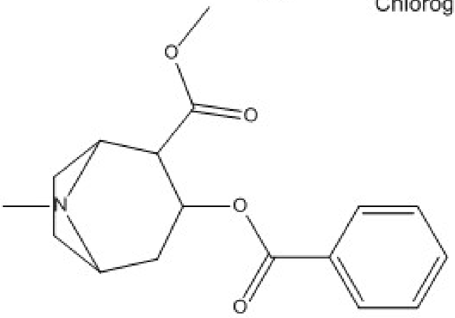

Cocaine

28

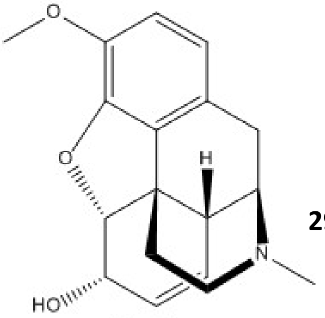

29

Codeine

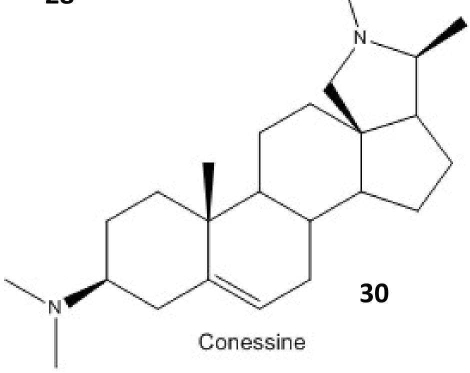

30

Conessine

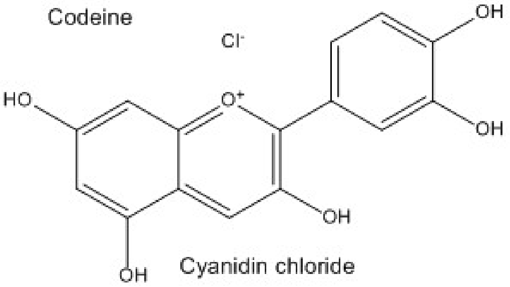

Cyanidin chloride

31

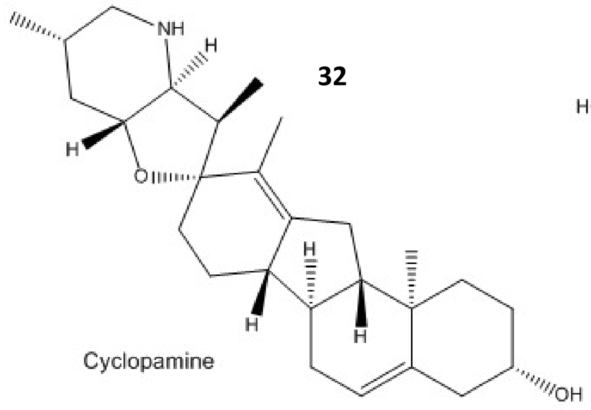

32

Cyclopamine

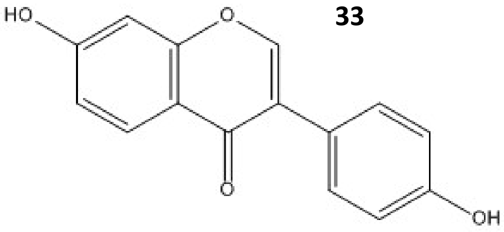

33

Daidzein

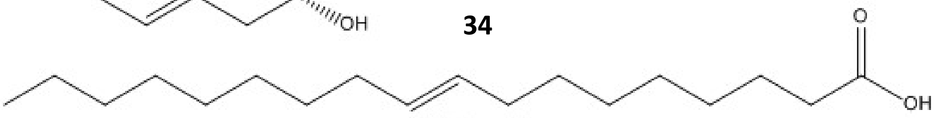

34

Elaidic acid

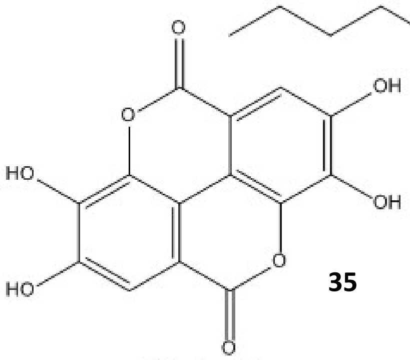

35

Ellagic acid

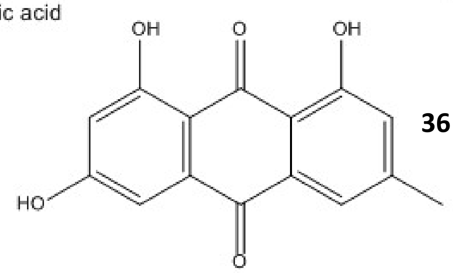

36

Emodin

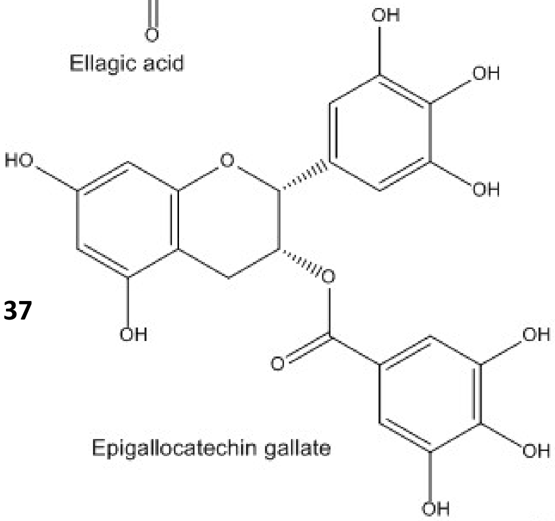

37

Epigallocatechin gallate

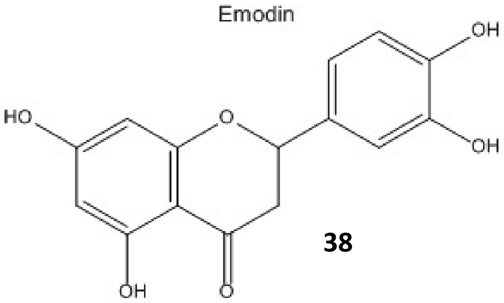

38

Eriodictyol

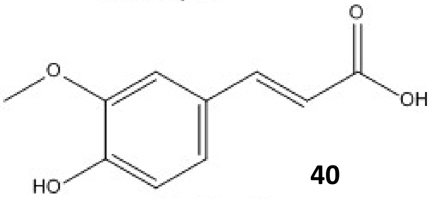

40

Ferulic acid

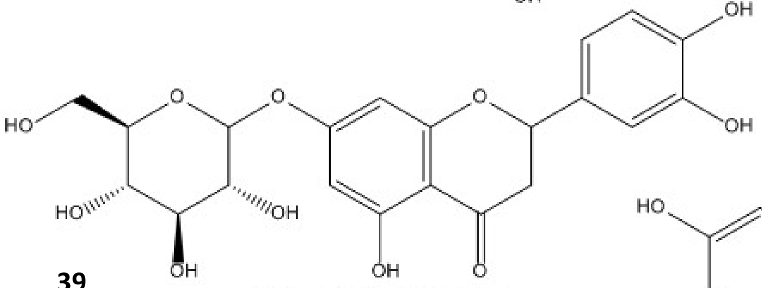

39

Eriodictyol 7-O-glucoside

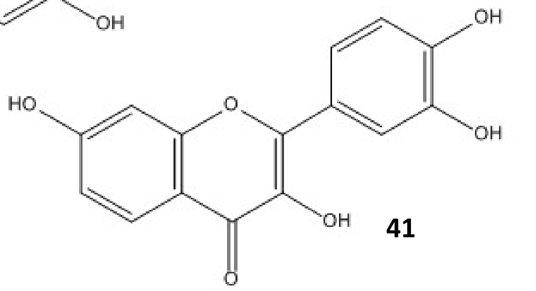

41

Fisetin

42

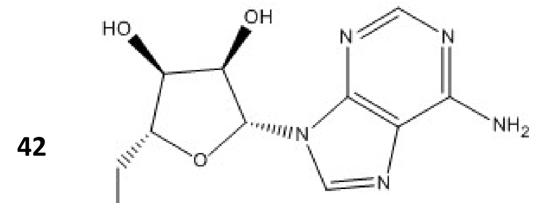

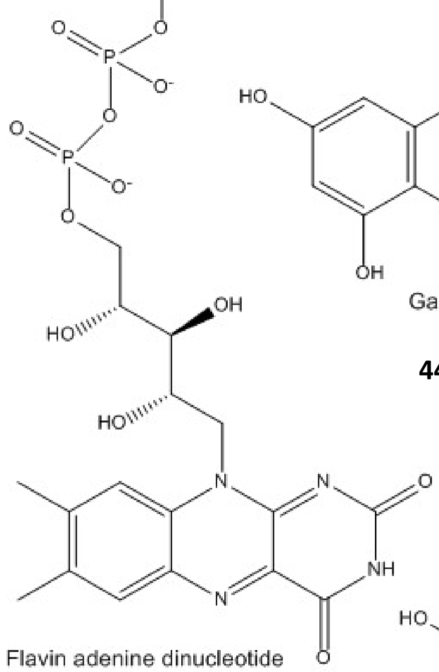

Flavin adenine dinucleotide

43

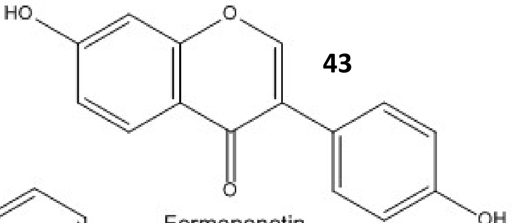

Formononetin

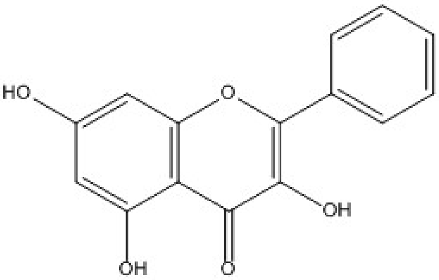

Galangin

44

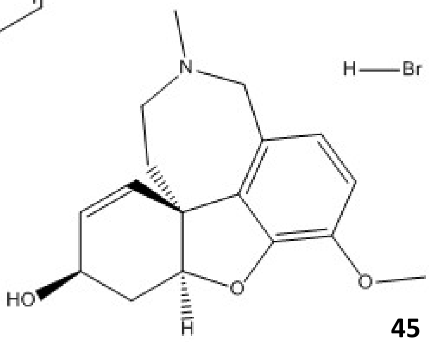

45

Galanthamine hydrobromide

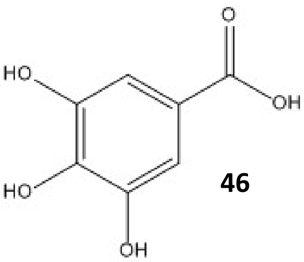

46

Gallic acid

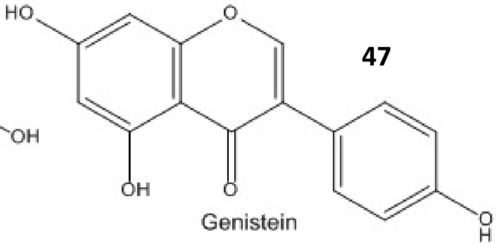

47

Genistein

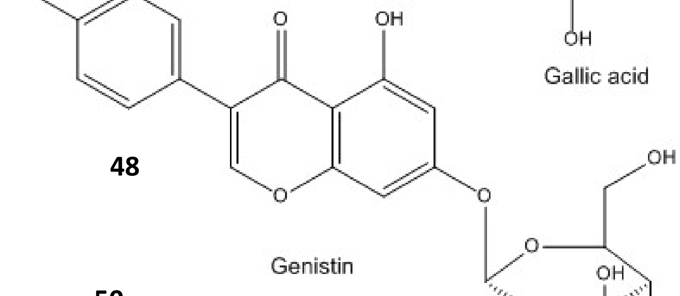

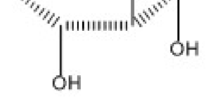

48

Genistin

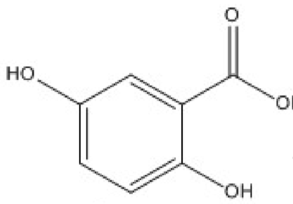

49

Gentisic acid

50

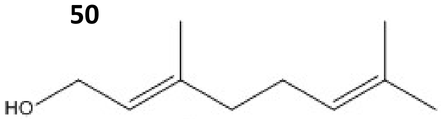

Geraniol

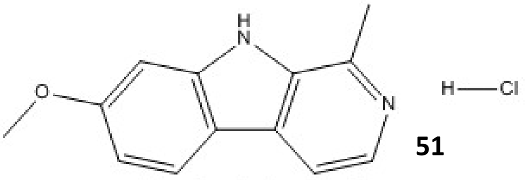

51

Harmine hydrochloride

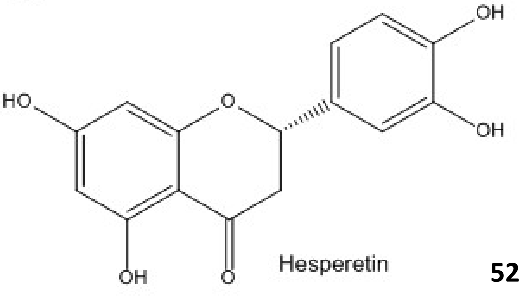

Hesperetin

52

53

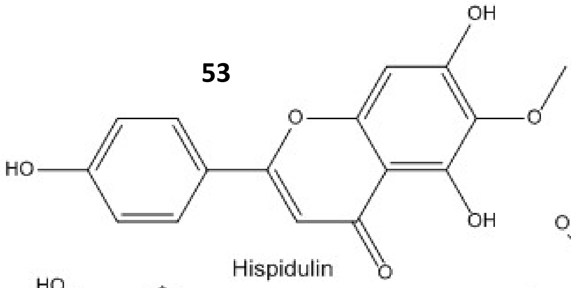

Hispidulin

54

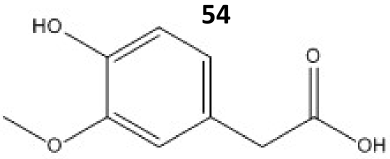

Homovanillic acid

55

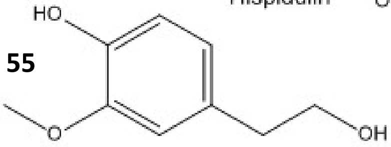

Homovanillyl alcohol

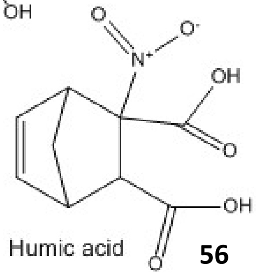

Humic acid **56**

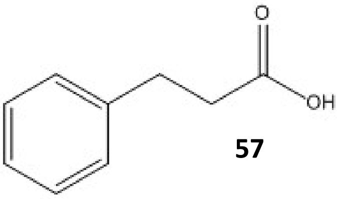

Hydrocinnamic acid **57**

58

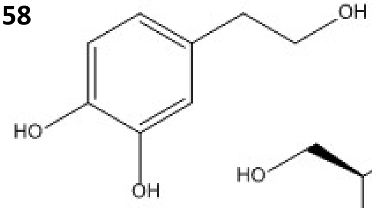

Hydroxytyrosol

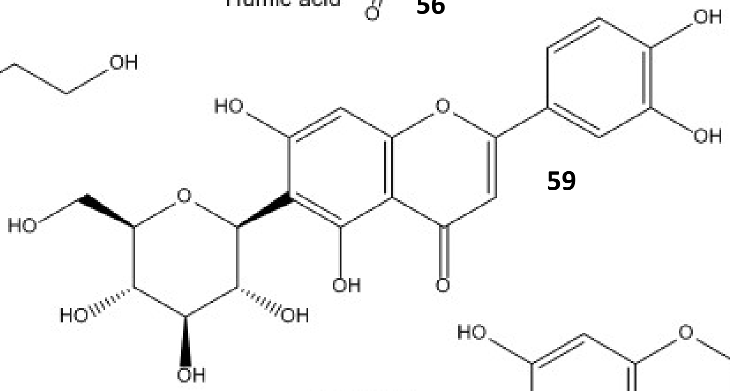

Isoorientin **59**

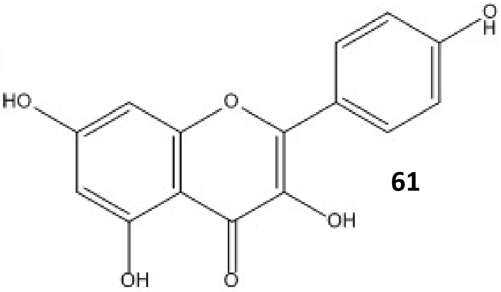

Kaempferol **61**

60

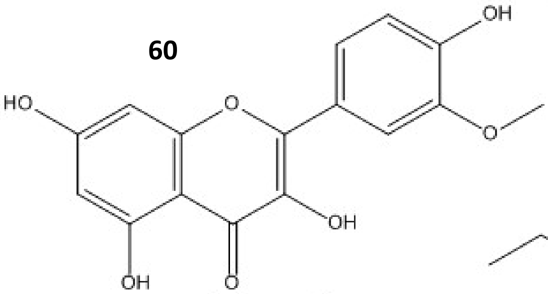

Isorhamnetin

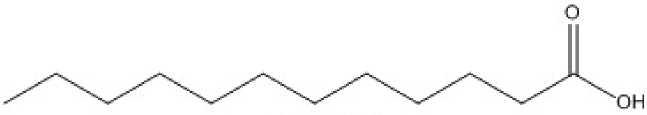

Lauric acid **62**

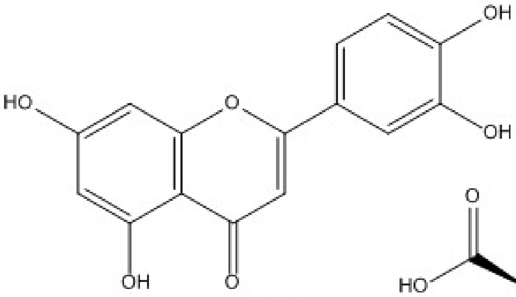

Luteolin

63

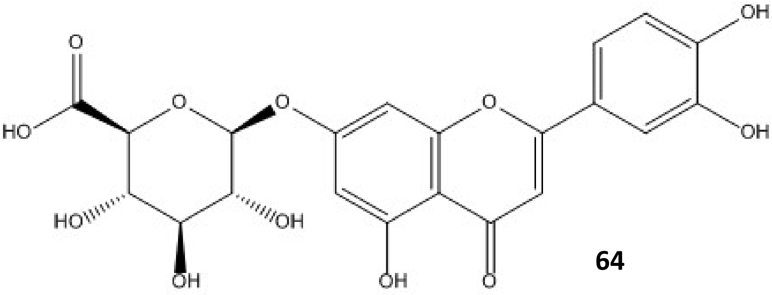

Luteolin 7-O-B-D-Glucuronide **64**

65

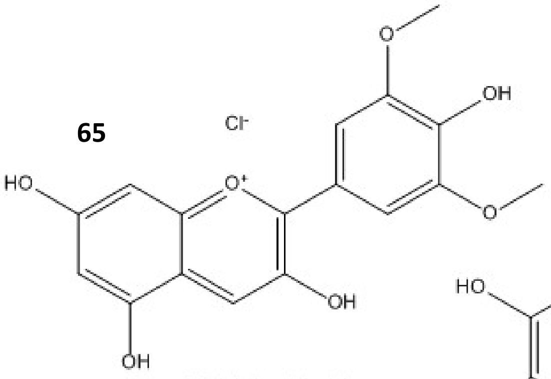

Malvidin chloride

66

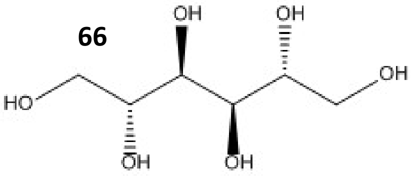

Mannitol

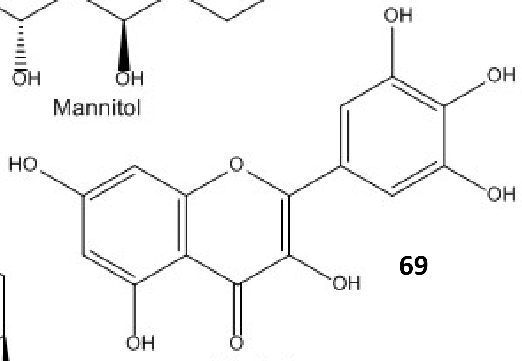

69

Myricetin

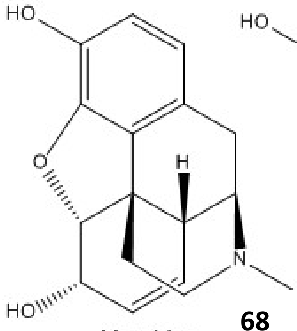

68

Morphine

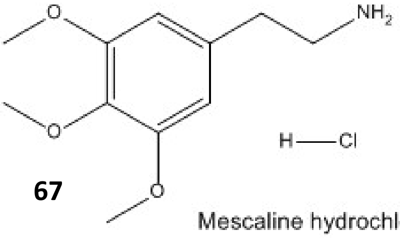

67

Mescaline hydrochloride

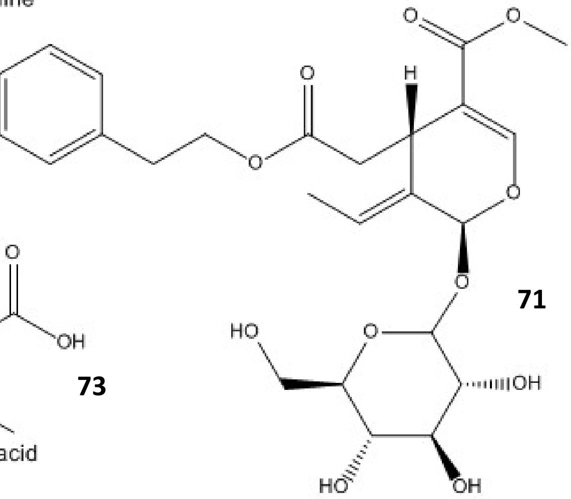

71

Oleuropein

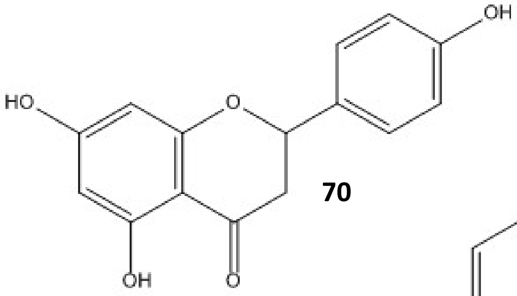

70

Naringenin

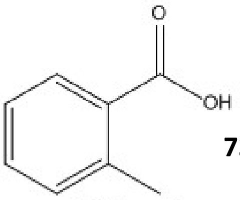

73

o-Toluic acid

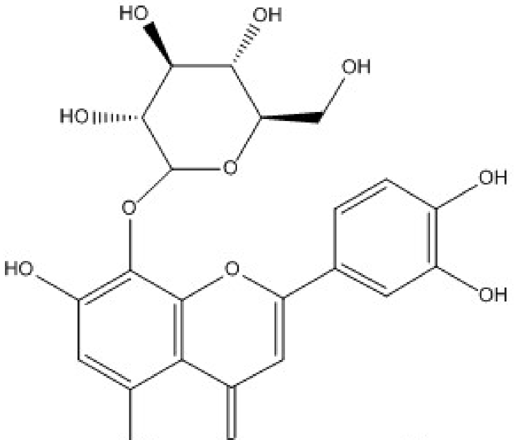

72

Orientin

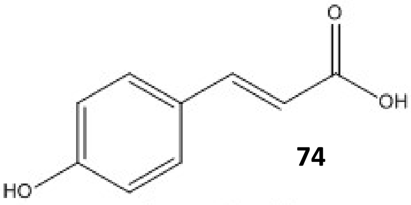

74

p-Coumaric acid

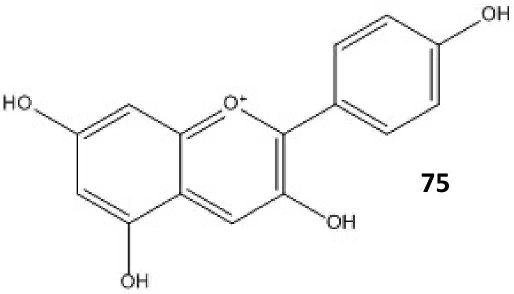

75

Pelargonidin

76

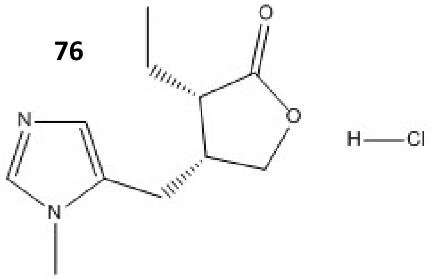

Pilocarpine hydrochloride

77

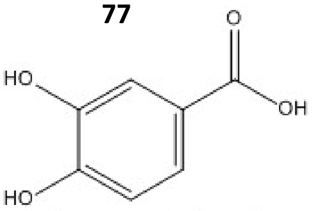

Protocatechuic acid

78

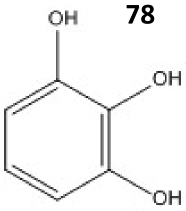

Pyrogallol

79

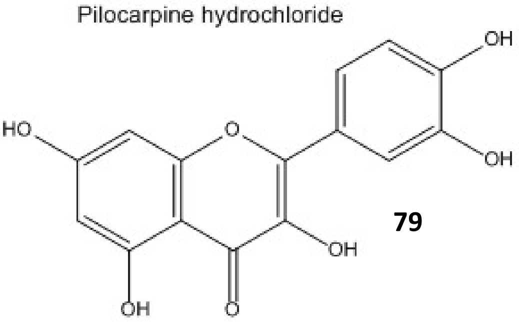

Quercetin

80

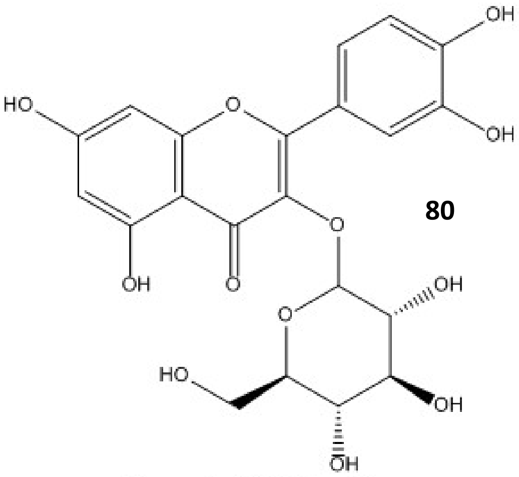

Quercetin-3-O-glucoside

81

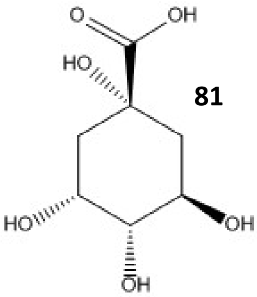

Quinic acid

82

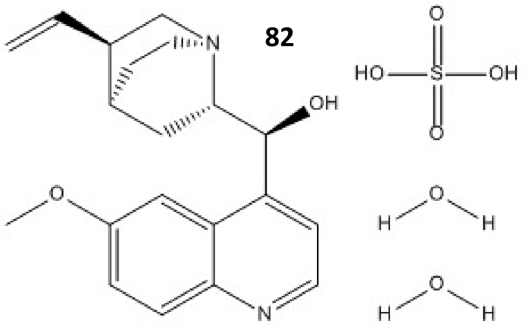

Quinidine sulfate dihydrate

83

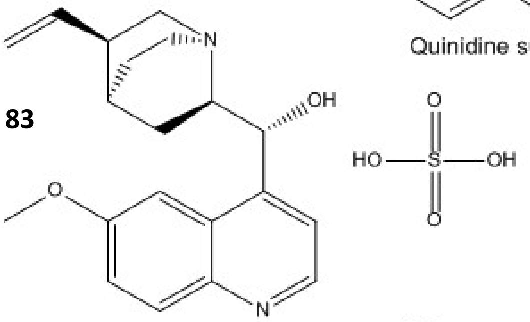

Quinine bisulfate

84

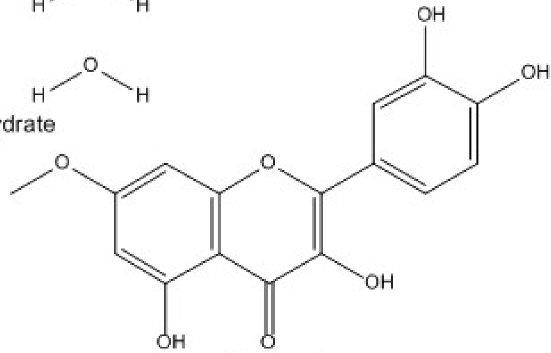

Rhamnetin

85

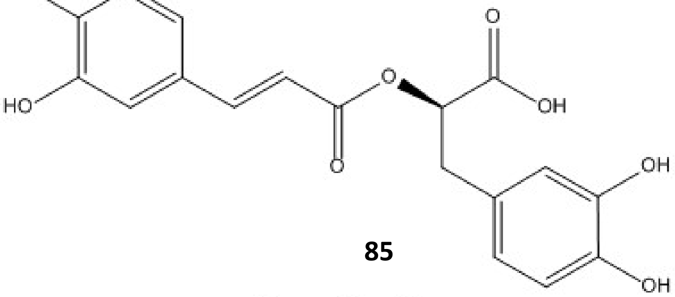

Rosmarinic acid

86

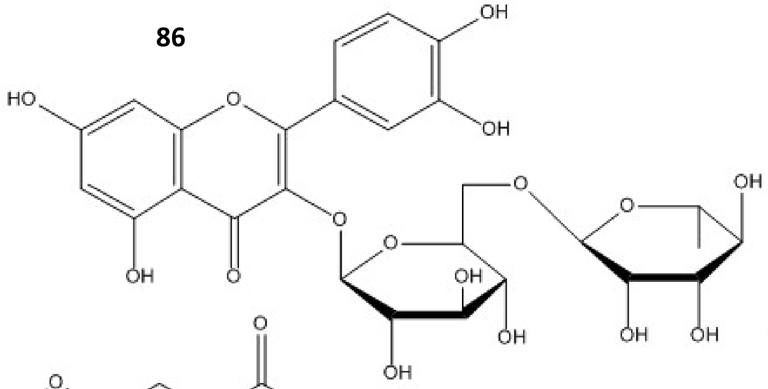

Rutin

87

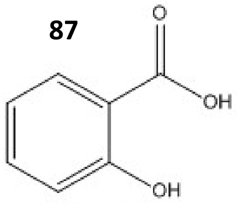

Salicylic acid

88

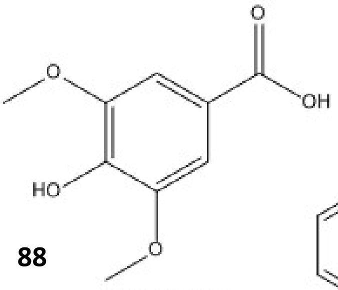

Syringic acid

89

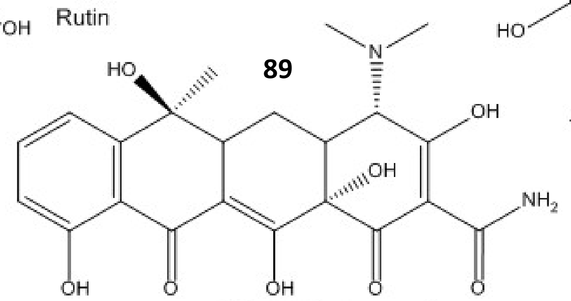

Tetracycline

90

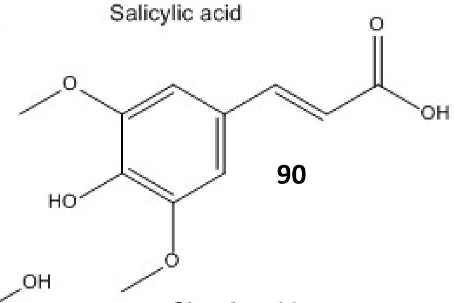

Sinapic acid

91

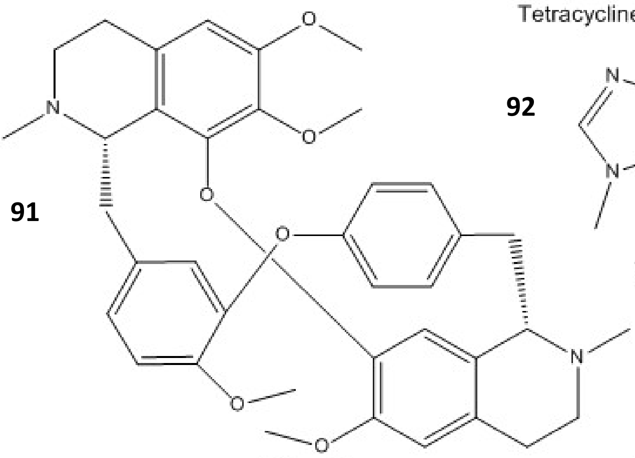

Tetrandrine

92

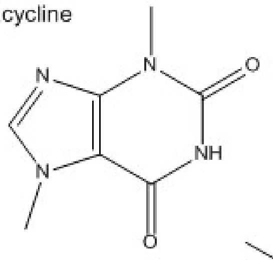

Theobromine

93

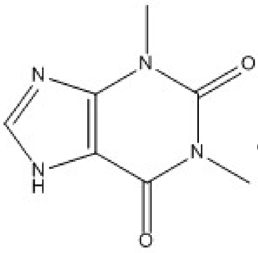

Theophylline

94

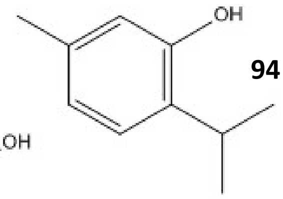

Thymol

95

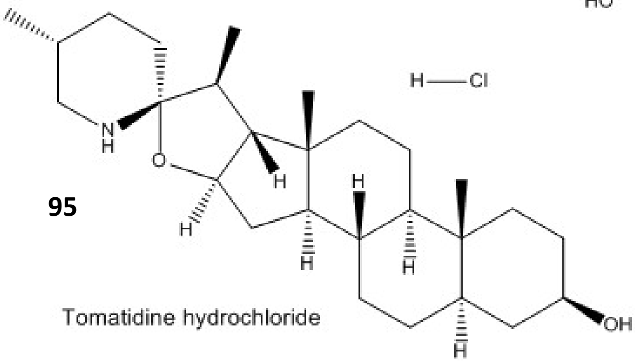

Tomatidine hydrochloride

96

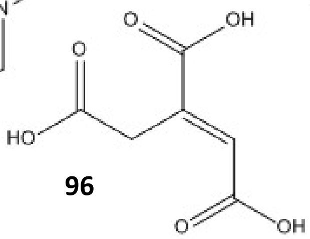

trans-Aconitic acid

97

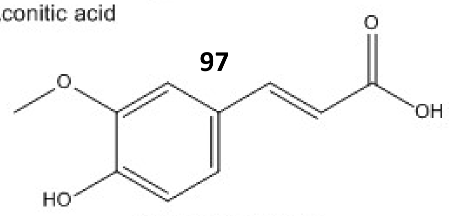

trans-Ferulic acid

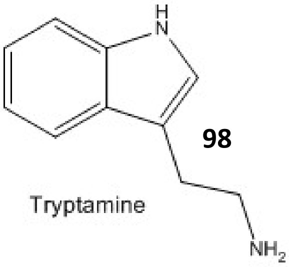

98 Tryptamine

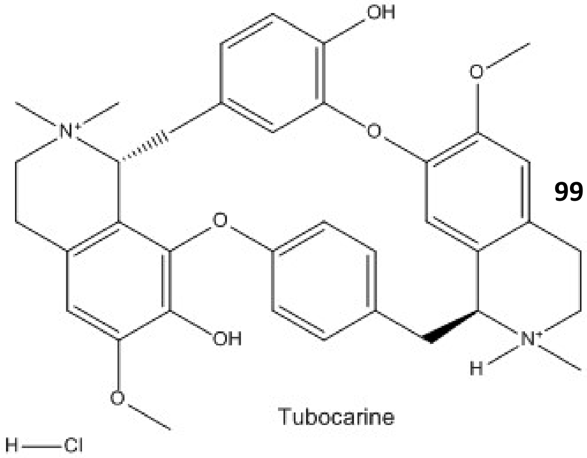

99 Tubocarine

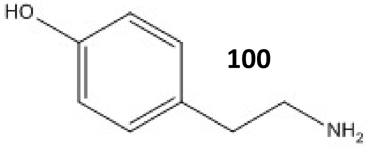

100 Tyramine hydrochloride

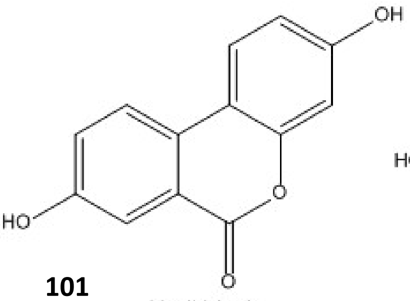

101 Urolithin A

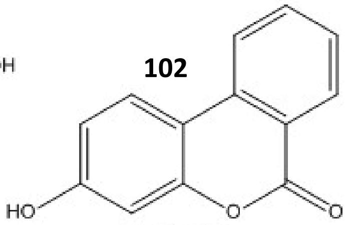

102 Urolithin B

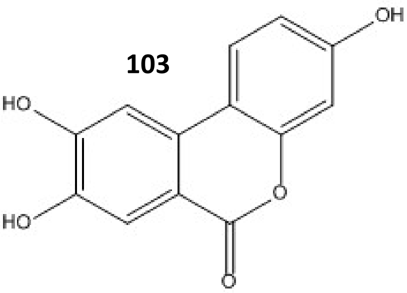

103 Urolithin C

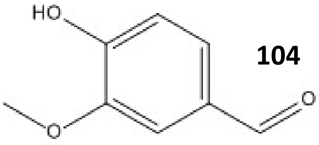

104 Vanillin

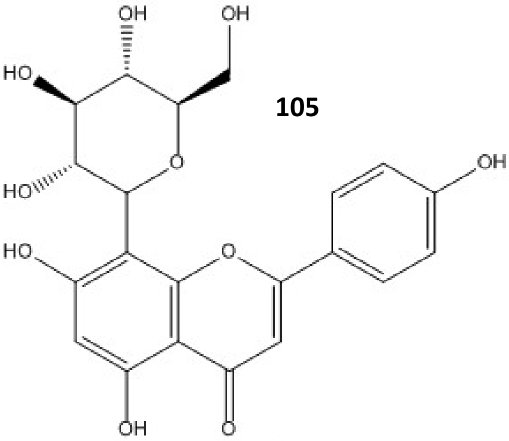

105 Vitexin

Supplementary Figure 6 | Polyphenols show a range of affinities for hydroxyapatite (HAP) linked to chemical structure. Chemical structure for 105 polyphenolic compounds used in binding studies.

Bone sample	Species	Animal breed	Feeding study	Notes
6070116	Sus scrofa	Duroc sires on Large White X German Landrace	Yes	Part of feeding experiment with controlled acorn supplementation of the diet.
6070125	Sus scrofa	Duroc sires on Large White X German Landrace	Yes	Part of feeding experiment with controlled acorn supplementation of the diet.
6070115	Sus scrofa	Duroc sires on Large White X German Landrace	Yes	Part of feeding experiment with controlled acorn supplementation of the diet.
1196353	Sus scrofa	Duroc sires on Large White X German Landrace	Yes	Part of feeding experiment with controlled acorn supplementation of the diet.
1196497	Sus scrofa	Duroc sires on Large White X German Landrace	Yes	Part of feeding experiment with controlled acorn supplementation of the diet.
PC-1	Sus scrofa	Domestic pig of unknown breed.	No	Used as a positive control for dietary acorn supplementation.
NC-1	Sus scrofa	Domestic pig of unknown breed.	No	Used as a negative control for dietary acorn supplementation.

Supplementary Table 1 | Pigs used in the Sardinian acorn-supplement feeding study.

No.	Compound ID	RT (min.)	$[M-H]^-$	Formula
1	2-(4-hydroxyphenyl)ethanol	5.07	137.0601	C8H10O2
2	2,4,6-Tris(2-pyridyl)-s-triazine	6.58	311.1043	C18H12N6
3	Aconitine	7.27	644.3064	C34H47NO11
4	Ajmalicine	4.90	351.1701	C21H24N2O3
5	Alizarin	3.54	239.0350	C14H8O4
6	Amentoflavone	8.17	537.0820	C30H18O10
7	Anabasine Hydrochloride	16.81	161.1072	C10H14N2
8	Apigenin	13.3	269.0448	C15H10O5
9	Apigenin-7-O-Glucuronide	6.85	445.0764	C21H18O11
10	Apigenin-7-O-neohesperidoside	5.33	577.1556	C27H30O14
11	Arachidic Acid	13.3	311.2954	C20H40O2
12	Baicalein	7.40	269.0448	C15H10O5
13	Benzoic acid	5.27	121.0288	C7H6O2
14	Berberine Chloride	5.11	370.0850	C20H18NO4Cl
15	Biochanin A	8.41	283.0599	C16H12O5
16	Caffeic acid	5.32	179.0343	C9H8O4
17	Caffeine	7.53	193.0729	C8H10N4O2
18	Capsaicin	8.71	304.1911	C18H27NO3
19	Carmine	2.30	491.0831	C22H20O13
20	Carvacrol	11.9	149.0959	C10H14O
21	Castanospermine	0.72	188.0916	C8H15NO4
22	Catechin hydrate	4.97	289.0710	C15H14O6
23	Catharanthine	6.10	335.1752	C21H24N2O2
24	Chicoric acid	4.19	473.0718	C22H18O12
25	Chlorogenic acid	3.50	353.0871	C16H18O9
26	Chrysin	8.48	253.0499	C15H10O4
27	Cinchonidine	8.69	293.1647	C19H22N2O
28	Cocaine	5.39	302.1385	C17H21NO4
29	Codeine	6.55	298.1436	C18H21NO3
30	Conessine	4.87	355.3106	C24H40N2
31	Cyanidin Chloride	5.65	321.0159	C15H11O6
32	Cyclopamine	7.78	410.3052	C27H41NO2
33	Daidzein	6.02	253.0499	C15H10O4
34	Elaidic Acid	12.9	281.2484	C18H34O2
35	Ellagic acid	5.33	300.9983	C14H6O8
36	Emodin	8.66	269.0454	C15H10O5
37	Epigallocatechin gallate	3.55	457.0769	C22H18O11
38	Eriodictyol	5.65	287.0554	C15H12O6
39	Eriodictyol-7-O-glucoside	4.50	449.1082	C21H22O11
40	Ferulic Acid	4.72	193.0505	C10H10O4
41	Fisetin	6.55	285.0397	C15H10O6
42	Flavin Adenine Dinucleotide	3.62	784.1497	C27H33P2N9O15
43	Formononetin	7.61	267.0650	C16H12O4
44	Galangin	10.3	269.0448	C15H10O5
45	Galanthamine Hydrobromide	0.84	286.1436	C17H21NO3
46	Gallic acid	1.28	169.0135	C7H6O5
47	Genistein	6.72	269.0448	C15H10O5
48	Genistin	4.71	431.0976	C21H20O10
49	Gentisic acid	3.55	153.0186	C7H6O4
50	Geraniol	6.15	153.1272	C10H18O
51	Harmine Hydrochloride	8.48	211.0864	C13H12N2O
52	Hesperetin	6.60	301.0710	C16H14O6
53	Hispidulin	7.28	299.0554	C16H12O6
54	Homovanilic acid	3.96	181.0499	C9H10O4
55	Homovanillyl Alcohol	12.1	167.0712	C9H12O3
56	Humic Acid	8.45	225.0138	C9H8Na2O4

No.	Compound ID	RT (min.)	$[M\text{-}H]^-$	Formula
57	Hydrocinnamic acid	6.08	149.0601	C9H10O2
58	Hydroxytyrosol	2.85	153.0555	C8H10O3
59	Isoorientin	4.51	447.092	C21H20O11
60	Isorhamnetin	7.80	315.0503	C16H12O7
61	Kaempferol	6.55	285.0397	C15H10O6
62	Lauric Acid	11.3	199.1702	C12H24O2
63	Luteolin	6.55	285.0397	C15H10O6
64	Luteolin-7-O-β-D-Glucuronide	4.92	461.0718	C21H18O12
65	Malvidin Chloride	5.86	365.0432	C17H15O7
66	Mannitol	0.85	181.0718	C6H14O6
67	Mescaline HCl	8.48	210.1123	C11H17NO3
68	Morphine	7.28	284.1279	C17H19NO3
69	Myricetin	5.45	317.0296	C15H10O8
70	Naringenin	4.97	271.0605	C15H12O5
71	Oleuropein	2.67	539.1770	C25H32O13
72	Orientin	4.51	447.0920	C21H20O11
73	o-Toluic Acid	3.9	135.0439	C8H802
74	p-Coumaric acid	6.37	163.0393	C9H8O3
75	Pelargonidin	4.57	270.0526	C15H11O5
76	Pilocarpine Hydrochloride	8.48	207.1126	C11H16N2O2
77	Protocatechuic acid	4.25	153.0186	C7H6O4
78	Pyrogallol	1.46	125.0237	C6H6O3
79	Quercetin	6.26	301.0347	C15H10O7
80	Quercetin-3-O-glucoside	5.07	463.0875	C21H20O12
81	Quinic acid	0.89	191.0554	C7H12O6
82	Quinidine Sulfate Dihydrate	5.86	647.3590	C40H48N4O4
83	Quinine Bisulfate	4.71	323.1758	C20H24N2O2
84	Rhamnetin	7.80	315.0503	C16H12O7
85	Rosmarinic acid	5.19	359.0765	C18H16O8
86	Rutin	11.3	609.1454	C27H30O16
87	Salicylic acid	5.43	137.0237	C7H6O3
88	Sinapic acid	4.72	223.0605	C11H12O5
89	Syringic acid	5.19	197.0448	C9H10O5
90	Tetracycline	3.75	443.1453	C22H24N2O8
91	Tetrandrine	5.76	621.2957	C38H42N2O6
92	Theobromine	3.20	179.0562	C7H8N4O2
93	Theophylline	3.31	179.0562	C7H8N4O2
94	Thymol	11.9	149.2096	C10H14O
95	Tomatidine (hydrochloride)	9.25	414.3365	C27H45NO2
96	trans-Ferulic acid	4.72	193.0494	C10H10O4
97	trans-Aconitic acid	1.15	173.0084	C6H6O6
98	Tryptamine	14.3	159.0915	C10H12N2
99	Tubocurarine Hydrochloride Penta-hydrate	4.57	609.2963	C37H42N2O6
100	Tyramine Hydrochloride	1.07	136.0755	C8H11NO
101	Urolithin A	5.96	227.0343	C13H8O4
102	Urolithin B	7.03	211.0393	C13H8O3
103	Urolithin C	6.59	243.0292	C13H8O5
104	Vanillin	4.28	151.0393	C8H8O3
105	Vitexin	4.71	431.0976	C21H20O10

Supplementary Table 2 | Authentic compound standards used for the identification of plant-derived compounds in bone extracts.

Compound	Formula	RT (min)	m/z ppm error	Percentage adsorption to HAP	Standard error percentage
Carmine	C22H20O13	2.29	2.4843	100	3.02
Chlorogenic acid	C16H18O9	1.89	1.92587	99	0.74
Flavin Adenine Dinucleotide	C27H33N9O15P2	6.31	2.49952	99	5.57
Chicoric acid	C22H18O12	2.67	-0.42277	99	8.84
Quinic acid	C7H12O6	0.93	1.04681	97	1.53
trans-Aconitic acid	C6H6O6	1.04	2.08081	92	3.09
Tetracycline	C22H24N2O8	1.96	-0.11283	89	1.61
Mannitol	C6H14O6	0.85	1.98816	87	2.67
Luteolin-7-O-β-D-Glucuronide	C21H18O12	2.5	-0.5639	78	5.60
Orientin	C21H20O11	2.19	0.62627	76	4.17
p-Coumaric acid	C9H8O3	2.42	0.49068	58	3.44
Quercetin-3-O-glucoside	C21H20O12	2.56	-0.06478	56	6.21
Ellagic acid	C14H6O8	2.71	-0.09834	52	2.41
Rutin	C27H30O16	2.67	0.68604	52	7.08
Urolithin A	C13H8O4	2.94	1.06592	52	7.87
Rosmarinic acid	C18H16O8	2.59	0.25259	49	7.13
Protocatechuic acid	C7H6O4	2.85	2.10431	47	2.22
Vitexin	C21H20O10	3.48	1.29901	47	6.90
Benzoic acid	C7H6O2	2.82	4.40141	46	12.03
Oleuropein	C25H32O13	2.67	0.93494	46	5.47
Isoorientin	C21H20O11	2.37	-0.43705	46	3.83
Epigallocatechin gallate	C22H18O11	4.13	0.53492	45	3.85
trans-Ferulic acid	C10H10O4	2.45	1.46127	44	4.61
Hydrocinnamic acid	C9H10O2	3.06	3.8434	43	9.69
Apigenin-7-O-neohesperidoside	C27H30O14	2.77	3.3244	42	1.33
Alizarin	C14H8O4	3.56	-0.28448	40	3.20
Gentisic acid	C7H6O4	2.17	2.88852	39	2.31
Eriodictyol-7-O-glucoside	C21H22O11	2.4	0.98996	39	1.05
Lauric Acid	C12H24O2	4.34	2.87844	33	7.09
Syringic acid	C9H10O5	2.29	1.55649	33	1.27
Capsaicin	C18H27NO3	3.61	0.35175	29	8.67
Caffeic acid	C9H8O4	2.18	4.8147	23	5.82
Salicylic acid	C7H6O3	2.9	4.06789	22	3.26
Urolithin C	C13H8O5	2.67	0.19175	22	4.25
o-Toluic Acid	C8H8O2	3.07	2.61246	20	6.97
Amentoflavone	C30H18O10	3.39	4.35668	20	7.41
Baicalein	C15H10O5	3.33	-0.16094	20	1.72
Fisetin	C15H10O6	5.65	0.07473	17	2.90
Ferulic Acid	C10H10O4	2.47	0.01088	17	1.59
Sinapic acid	C11H12O5	2.45	0.29902	17	2.29
Hesperetin	C16H14O6	3.24	2.39611	16	8.33
Luteolin	C15H10O6	3.07	2.88135	15	0.80
Ajmalicine	C21H24N2O3	2.44	1.89622	15	5.14
Myricetin	C15H10O8	2.74	3.12431	14	3.57
Isorhamnetin	C16H12O7	3.25	1.35184	13	1.87
Pyrogallol	C6H6O3	1.63	2.13798	12	0.82
Kaempferol	C15H10O6	3.21	-0.38135	12	1.65
Catechin hydrate	C15H14O6	1.8	0.35078	9	4.88
Rhamnetin	C16H12O7	3.4	0.20917	8	3.72
Gallic acid	C7H6O5	1.08	1.28155	6	3.12
Hispidulin	C16H12O6	3.28	1.40877	5	1.64
Eriodictyol	C15H12O6	2.82	0.59675	5	2.99
Urolithin B	C13H8O3	3.4	1.1249	4	1.98
Chrysin	C15H10O4	3.63	2.77454	3	2.86
Vanillin	C8H8O3	2.34	0.57865	2	3.36
Galangin	C15H10O5	4.17	1.47447	2	0.95

Supplementary Table 3 | Shows the extent to which a wide range of polyphenols bind to hydroxyapatite (HAP), the mineral component of bone. 100% binding (carmine for example) was determined by complete loss of detectable polyphenol when mixed with HAP (from a standard solution concentration/weight of HAP). The results show that there is variability from 100% to 0% binding. No strong structural or chemical patterns were observed to be associated with strong binding, however, multiple adjacent hydroxyl groups on a single ring with a single or

Acorns [%] in the diet<br>Chemical composition of the diets	0 %	50 %
Dry matter [g/kg, as fed]	890	751
Crude ash [g/kg DM]	68.5	58.6
Crude protein [g/kg DM]	175	116
Crude fibre [g/kg DM]	45.0	95
Starch [g/kg DM]	390	410
Tannic acid equivalent [g/kg DM]	<1.0*	25.8
Dry matter [g/kg, as fed]	890	751

Supplementary Table 4 | The chemical composition of Sardinia experimental diets. *below limit of detection.

Parameters	Weeks of feeding	Growers	Finishers
(n. animals)	4	3	2
BM at slaughter (kg)		5.40±0.25	127±2.80
DM intake (g*kg $BM^{-1}d^{-1}$)		66.5±0.80	23.6±1.72
TAE intake (mg*kg $BM^{-1}d^{-1}$)		1717±13	610±0.01
(n. animals)	4	3	2
BM at slaughter (kg)		5.40±0.25	127±2.80
DM intake (g*kg $BM^{-1}d^{-1}$)		66.5±0.80	23.6±1.72

Supplementary Table 5 | Average daily intakes of dry matter (DM) and tannic acid equivalent (TAE) (M±SE) in acorn fed pigs involved in the Sardinian pig feeding experimental trial.
